# Concordance of CSF measures of Alzheimer's pathology with amyloid PET status in a preclinical cohort: A comparison of Lumipulse and established immunoassays

**DOI:** 10.1002/dad2.12131

**Published:** 2021-02-06

**Authors:** Ashvini Keshavan, Henrietta Wellington, Zhongbo Chen, Ayesha Khatun, Miles Chapman, Melanie Hart, David M. Cash, William Coath, Thomas D. Parker, Sarah M. Buchanan, Sarah E. Keuss, Matthew J. Harris, Heidi Murray‐Smith, Amanda Heslegrave, Nick C. Fox, Henrik Zetterberg, Jonathan M Schott

**Affiliations:** ^1^ Dementia Research Centre UCL Queen Square Institute of Neurology, University College London London UK; ^2^ UK Dementia Research Institute Fluid Biomarkers Laboratory UK DRI at University College London London UK; ^3^ Neuroimmunology and CSF Laboratory National Hospital for Neurology and Neurosurgery London UK; ^4^ Department of Neuroinflammation UCL Queen Square Institute of Neurology, University College London London UK; ^5^ Clinical Neurochemistry Laboratory, Department of Psychiatry and Neurochemistry Institute of Neuroscience and Physiology, The Sahlgrenska Academy at University of Gothenburg, Sahlgrenska University Hospital Mölndal Sweden

**Keywords:** amyloid, cerebrospinal fluid, Lumipulse, positron emission tomography, tau

## Abstract

**INTRODUCTION:**

We assessed the concordance of cerebrospinal fluid (CSF) amyloid beta (Aβ) and tau measured on the fully automated Lumipulse platform with pre‐symptomatic Alzheimer's disease (AD) pathology on amyloid positron emission tomography (PET).

**METHODS:**

In 72 individuals from the Insight 46 study, CSF Aβ40, Aβ42, total tau (t‐tau), and phosphorylated tau at site 181 (p‐tau181) were measured using Lumipulse, INNOTEST, and Meso Scale Discovery (MSD) assays and inter‐platform Pearson correlations derived. Lumipulse Aβ42 measures were adjusted to incorporate standardization to certified reference materials. Logistic regressions and receiver operating characteristics analysis generated CSF cut‐points optimizing concordance with ^18^F‐florbetapir amyloid PET status (n = 63).

**RESULTS:**

Measurements of CSF Aβ, p‐tau181, and their ratios correlated well across platforms (r 0.84 to 0.94, *P* < .0001); those of t‐tau and t‐tau/Aβ42 correlated moderately (r 0.57 to 0.79, *P* < .0001). The best concordance with amyloid PET (100% sensitivity and 94% specificity) was afforded by cut‐points of 0.075 for Lumipulse Aβ42/Aβ40, 0.087 for MSD Aβ42/Aβ40 and 17.3 for Lumipulse Aβ42/p‐tau181.

**DISCUSSION:**

The Lumipulse platform provides comparable sensitivity and specificity to established CSF immunoassays in identifying pre‐symptomatic AD pathology.

## 1 BACKGROUND

Cerebrospinal fluid (CSF) amyloid beta (Aβ) and tau, and quantification of cortical amyloid burden by positron emission tomography (PET) remain among the best‐established biomarkers of Alzheimer's disease (AD). Guidelines for their use in the clinical setting include the UK National Institute of Clinical Assessment Guideline 2018,^1^ and the Alzheimer's Association's appropriate use criteria for CSF testing^2^ and for amyloid PET.^3^ Biomarkers are key components of research criteria for AD, which is defined by the presence of AD pathology even in asymptomatic individuals,^4^ and are widely used as inclusion criteria and outcome measures for clinical trials.

A decrease in CSF concentration of soluble Aβ1–42 (Aβ42) peptide is one of the earliest changes in preclinical AD,^5^, ^6^, ^7^ likely reflecting the aggregation and deposition of Aβ into plaques in the brain.^8^ CSF Aβ42/Aβ40 ratio has consistently shown better diagnostic value for AD than Aβ42 alone,^9^ perhaps compensating for individual differences in the total production of Aβ and CSF turnover.^10^ The Aβ42/Aβ40 ratio has also been found to mitigate the adsorption‐related effects of low sample storage volume (< 1 mL) on measurements of Aβ concentrations by different platforms.^11^, ^12^

CSF Aβ42 has a high concordance of 89% to 92% with amyloid PET;^13^, ^14^ this is further improved when using CSF Aβ42/Aβ40 ratio, to 94% to 98%.^14^ Both reduced CSF Aβ42/Aβ40 ratio^15^ and increased uptake of amyloid PET tracers including 18F‐florbetapir^16^ have been shown to correlate with neuropathologically confirmed cerebral Aβ deposition.

Multiple analytical platforms are used for measuring core CSF AD biomarkers, for example, INNOTEST (Fujirebio) provides clinically validated enzyme‐linked immunosorbent assays (ELISA) for Aβ42, total tau (t‐tau), and phosphorylated tau at site 181 (p‐tau181); the Meso Scale Discovery (MSD) Aβ triplex electrochemiluminescence assay simultaneously measures Aβ38, Aβ40, and Aβ42. However, despite efforts to standardize biomarker measurements among multiple platforms and laboratories,^17^ differences in absolute values among platforms and coefficients of variation remain high, hampering the development of universal cut‐points for use in clinical settings. Therefore, there is a drive toward validating fully automated platforms that reduce manual steps as a source for variation. One of these automated platforms is the Lumipulse G system (Fujirebio), on which chemiluminescent immunoassays for Aβ40, Aβ42, t‐tau, and p‐tau181 have been developed, using the same antibodies as the INNOTEST ELISAs.

Recent studies directly comparing measurements by Lumipulse with INNOTEST ELISAs have shown good concordance between the two platforms but reduced intra‐ and inter‐assay variability on the Lumipulse.^18^, ^19^, ^20^, ^21^, ^22^ However, systematic differences in absolute CSF Aβ42 concentrations between Lumipulse and INNOTEST platforms have been observed,^18^, ^19^ with one study reporting 27% lower concentrations measured by INNOTEST compared to Lumipulse.^18^ When assessing the diagnostic accuracy of Lumipulse CSF Aβ and tau in classifying clinical AD from cognitively asymptomatic controls, Lumipulse ratios of Aβ42/Aβ40, Aβ42/t‐tau, and Aβ42/p‐tau181 were found to have a higher diagnostic accuracy than individual markers.^20^, ^21^

Other studies assessed the ability of Lumipulse assays to differentiate clinical AD from non‐AD neurological conditions. No significant difference in diagnostic accuracy has been shown between Lumipulse and INNOTEST assays;^19^ again, compared to using individual biomarkers, the Lumipulse Aβ42/t‐tau ratio,^19^ and the Aβ42/Aβ40 ratio^23^ showed improved diagnostic performance.

A few studies have assessed the concordance of CSF Aβ and tau with amyloid PET.^13^, ^24^, ^25^ Janelidze et al.^13^ investigated individuals with mild cognitive complaints, comparing concordance of CSF Aβ42, Aβ40, and t‐tau with visual amyloid PET status across five CSF assay platforms. Newer immunoassays, including a modified INNOTEST and Lumipulse assays, showed improved agreement with visual amyloid PET when using Aβ42/Aβ40 or Aβ42/t‐tau ratios (concordance 93% to 95%), compared to their respective Aβ42 assays (87%to 89%), but the classic INNOTEST Aβ42 assay gave a concordance of 92%. Spiked Aβ40 over a concentration range of 1 to 40 ng/mL led to progressive decrease in values of Aβ42 measured by the classic INNOTEST (with 60% reduction at the highest spiked concentration) and the MSD platform (with 20% at the highest spiked concentration), but not by the modified INNOTEST.^13^ Taken together, these results suggest that the classic INNOTEST assay exhibits some non‐specificity to Aβ42 measurement due to quenching of signal by Aβ40 levels.

When assessing CSF by Lumipulse in a mixed memory clinic cohort, Alcolea et al. also found a higher concordance with amyloid PET when using the CSF Aβ42/Aβ40 ratio (86%) than when using individual markers (76% to 84%).^24^ Kaplow et al. used Lumipulse CSF Aβ42 and t‐tau cut‐points to predict amyloid PET status in multiple cohorts and reported the best performance in all cohorts when using the t‐tau/Aβ42 ratio (concordance 85% to 95%).^25^

As yet, no single study has directly compared all four CSF Aβ and tau markers and ratios measured by the Lumipulse platform with established immunoassays and compared platforms according to concordance with amyloid PET in a preclinical setting. In the present study we extend the comparison of individual Lumipulse CSF Aβ40, Aβ42, t‐tau, and p‐tau181 markers to also include ratios, with direct comparison to the INNOTEST and MSD platforms. We supplement existing knowledge about the possible contribution of Aβ40 interference to differences in measurements of Aβ42 by evaluating all three platforms, and assess concordance of individual markers and ratios with amyloid PET imaging in a preclinical cohort.

## 2 METHODS

### 2.1 Participants and study design

Participants were from the second time point of Insight 46, the neuroscience substudy of the National Survey of Health and Development (NSHD, the 1946 British birth cohort), for which the study design has previously been described,^26^ and National Research Ethics Committee approval (REC reference 14/LO/1173; PI Schott) was obtained. Participants were population‐representative at their birth; although cognition was not used as a criterion for recruitment, we have previously shown, in a detailed examination of their representativeness,^27^ that those recruited to Insight 46 had better cognitive performance at age 69 than those from the wider NSHD who were not recruited to Insight 46. Participants provided written informed consent. As the second time point is ongoing, the CSF samples were from an interim dataset, collected from March 2018 to April 2019.

**RESEARCH IN CONTEXT**

1. Systematic review: The authors searched PubMed using the terms “Lumipulse AND (Alzheimer's OR amyloid).” Recent research has compared Lumipulse measurements of cerebrospinal fluid (CSF) amyloid beta (Aβ)42 and t‐tau with established manual immunoassays and assessed concordance of Lumipulse Aβ42, Aβ40, total tau (t‐tau), and phosphorylated tau at site 181 (p‐tau181) with amyloid positron emission tomography (PET). However, measurements of Aβ40 and p‐tau181 by Lumipulse and established immunoassays had not yet been compared, and concordance of Aβ42/Aβ40 and Aβ42/p‐tau181 ratios with amyloid PET had not been assessed in a preclinical cohort.
2. Interpretation: We compared Aβ42 measurements by three CSF platforms, and Aβ40, p‐tau181, and t‐tau across two platforms, in the same individuals from a British birth cohort aged 72 to 74. The highest concordance with ^18^F‐florbetapir PET was afforded by Lumipulse Aβ42/Aβ40 and Aβ42/p‐tau181 ratios, and Meso Scale Discovery Aβ42/Aβ40 ratio.
3. Future directions: Lumipulse quantification of Alzheimer's disease CSF biomarkers may allow for stratification of preclinical cohorts by amyloid status. As certified reference materials for these biomarkers are developed, cross‐platform cut‐point standardization may become achievable.

### 2.2 Lumbar punctures and pre‐analytical CSF processing

Exclusion criteria for lumbar puncture (LP) were clinical/neuroimaging safety concerns for raised intracranial pressure, known/suspected thrombocytopenia or coagulopathy, use of antiplatelet or anticoagulant medications (apart from aspirin 75 mg daily), congenital spinal malformation, lumbar fixation surgery, active skin inflammation overlying the proposed LP site, or lignocaine allergy. Participants were not instructed to fast, and LP was timed between 08:30 and 10:30 hrs. After local anesthesia with lignocaine, a 22G atraumatic spinal needle was used to collect up to 20 mL of CSF, without active withdrawal, into 2 × 10 mL polypropylene screw top containers (Sarstedt 62.610.018), which were transported on ice within 30 minutes to the laboratory. CSF was centrifuged at 1750 g for 10 minutes at 4°C and the supernatant placed in 0.5 mL aliquots into polypropylene screw top cryovials, to be stored at –80°C within 60 minutes of LP.

### 2.3 Imaging procedures

Dynamic ^18^F‐florbetapir (Amyvid) amyloid PET and magnetic resonance imaging (MRI) data were simultaneously acquired on a single Biograph mMR 3T PET/MRI scanner for all participants (Siemens Healthcare, Erlangen). The standardized uptake value ratio (SUVR) between a pre‐defined composite neocortical region of interest and an eroded white matter reference region was calculated, and a SUVR cut‐point of 0.61 was used to define amyloid PET status, as derived by mixture modeling generated at the first study time point.^28^

Eighty‐five percent of participants had their amyloid PET scan on the day before LP, but in the remaining 15%, either due to lack of tracer availability or participant choice, LP‐scan delay ranged between –13 and +110 days.

### 2.4 CSF assays

For each of the four analytes of interest, CSF measurements were undertaken using the Lumipulse platform and at least one other established immunoassay platform that uses manual steps in the measurement protocol (Table 1).

**TABLE 1:** Immunoassay platforms used and respective measured biomarkers. The ticks show the biomarkers measured by each platform

	Biomarker measured			
Platform	Aβ42	Aβ40	t‐tau	p‐tau181
Lumipulse	✓	✓	✓	✓
MSD	✓	✓		
INNOTEST	✓		✓	✓

Abbreviations: Aβ, amyloid beta; MSD, Mesoscale Discovery; p‐tau181, phosphorylated tau at site 181; t‐tau, total tau.

For measuring Aβ peptides, three assay platforms were used: INNOTEST β‐amyloid 1‐42 (Fujirebio) for Aβ42, Lumipulse G600II automated assay (Fujirebio) for Aβ42 and Aβ40, and MSD Multi‐spot Aβ 6E10 Triplex assay (Meso Scale Diagnostics) for Aβ42 and Aβ40. A single 500 µL aliquot of neat CSF was used to perform the INNOTEST and Lumipulse assays in parallel. INNOTEST required 25 µL/replicate for measuring Aβ42 alone. Lumipulse required 100 µL of dead volume, 50 µL/replicate for measuring Aβ42, and 40 µL/replicate for measuring Aβ40. A different aliquot of CSF from the same individuals was used to perform the MSD assay, with 15 µL/replicate of neat CSF (diluted 1:2 in assay diluent) used to measure all three peptides—Aβ38, Aβ40, and Aβ42—together.

T‐tau and p‐tau181 were measured in parallel on the INNOTEST and Lumipulse platforms, using the same aliquot of CSF. The INNOTEST hTau Ag assay required 25 µL/replicate and the INNOTEST phospho‐tau (181P) assay 75 µL/replicate. The Lumipulse assays required 100 µL of dead volume, 75 µL/replicate for measuring t‐tau, and 40 µL/replicate for measuring p‐tau181.

Samples were assayed after a single thaw to room temperature. On each platform, a single batch of reagents was used for all samples. Measurements by INNOTEST and MSD assays were performed in duplicate, and sample measurements accepted if coefficients of variation across duplicates were < 30%. Given that the Lumipulse platform required a larger total volume of CSF due to dead volume, measurements by Lumipulse were made once per sample.

Two run validation controls (provided with each assay kit) and two control CSF samples (provided by the Neuroimmunology and CSF Laboratory at the National Hospital for Neurology and Neurosurgery) with low and high values of the analyte(s) of interest were used. Intra‐run variation for the run validation controls and inter‐run variation using the control CSF samples are shown in Table S1 in supporting information. Measurements were performed according to the manufacturers’ instructions.

### 2.5 Aβ42 measurement standardization to certified reference material

After the measurements of Aβ peptides were performed in this study, certified reference materials (CRM) were produced for Aβ1‐42 and data have been published on the commutability of different assay platforms in measuring against these CRM.^29^ The values obtained by measuring Aβ42 on the Lumipulse platform were retrospectively adjusted to incorporate standardization against the CRM, using information kindly provided by Dr Nathalie Le Bastard (Fujirebio) for the Lumipulse assay.^30^ The INNOTEST and MSD calibrators have not been re‐standardized to the CRM, but the CRM has been shown to be commutable for assays on both platforms.^29^

### 2.6 Aβ40 interference

Investigation of Aβ40 interference with Aβ42 measurements is detailed in the supporting information.

### 2.7 Statistical analysis

All analyses used Stata v14.2 (Stata Corporation, College Station, Texas, USA). As individual biomarkers have a positively skewed distribution, log‐transformation was undertaken before assessing Pearson correlations between individual biomarker values across platforms. Such transformation was not required before assessing correlations between ratios. All individuals with available CSF data were included in correlation analyses.

Spearman correlation was used to assess the impact of spiking increasing concentrations of Aβ40 on measurements of Aβ42. Significant Aβ40 concentration‐dependent interference was shown if the correlation coefficient (rho) between measured Aβ42 and spiked Aβ40 concentration was significantly less than zero.

In the group with full CSF and amyloid PET data, differences in demographic characteristics between amyloid PET positive and negative groups were assessed using *t* tests for age at LP, and χ2 tests for sex (% male) and apolipoprotein E (*APOE*) ε4 carrier status (defined as % carrying one or two *APOE*4 alleles). Differences between groups in Mini‐Mental State Examination (MMSE) and measured biomarker values were assessed using Wilcoxon rank‐sum tests.

Logistic regression models with amyloid PET status as the outcome and CSF biomarkers or their ratios as predictors were used to perform receiver operating characteristics (ROC) analysis. The area under the ROC curve (AUC) was compared across biomarkers and platforms using De Long tests. Optimal CSF cut‐points for classifying amyloid PET positive versus negative individuals were ascertained using the Youden index.

## 3 RESULTS

### 3.1 Participant characteristics

Of 72 participants with CSF samples, 63 had full CSF and amyloid PET data. Of these, 71.4% were male and 22.4% carried one or two *APOE* ε4 alleles. Comparing those with full data with those excluded due to incomplete data, there were no significant differences in age, sex, *APOE* ε4 carrier status, MMSE, or any of the measured CSF biomarkers (Table S2 in supporting information).

Of the individuals included in the analyses against amyloid PET, 13 (20.6%) were PET–positive. Table 2 shows the demographic data and CSF biomarker values for the PET–negative and PET–positive groups. The PET–positive group were older than the PET–negative group (73.4 vs 72.5 years, *P* = .031) and had a higher percentage of *APOE* ε4 carriers (46.1 vs 16.3%, *P* = .022). All three platforms measured significantly lower (by 41% to 47%) CSF Aβ42 in PET–positive individuals (median concentration in pg/mL [interquartile range (IQR)]: Lumipulse PET–positive 711 [618, 923] vs PET–negative 1330 [955, 1633], *P* < .0001; MSD: 471 [350, 528] vs 801 [607, 942], *P* = .0001; INNOTEST 669 [560, 788] vs 1252 [1006, 1458], *P* < .0001). Similarly, the CSF Aβ42/Aβ40 ratio was significantly lower (by ≈50%) in PET–positive individuals (median ratio [IQR]: Lumipulse 0.050 [0.040, 0.062] vs 0.102 [0.094, 0.108], *P* < .0001; MSD 0.058 [0.047, 0.073]) vs 0.113 [0.105, 0.118, *P* <.0001]).

**TABLE 2:** Participant characteristics with respect to amyloid PET status

	All included in PET analysis n = 63 unless otherwise stated	Amyloid PET negative n = 50 unless otherwise stated	Amyloid PET positive n = 13 unless otherwise stated	*P*
Demographics				
Mean age at CSF sampling (SD), years	72.7 (1.3)	72.5 (0.3)	73.4 (2.8)	.031
Sex, % male	71.4	74.0	61.5	.376
*APOE* ε4 carrier status, % carrying one or two alleles	22.6 (n = 62)	16.3 (n = 49)	46.1	.022
Median MMSE (IQR)	29 (28, 30)	29 (28, 30)	29 (28, 29)	.730
LP‐scan interval >1day (%)	14.9	15.1	14.3	.940
Lumipulse platform results				
Median CSF Aβ40 (IQR), pg/mL	13193 (10528, 16376)	13323 (10377, 16047)	12968 (11323, 18221)	.262
Median CSF Aβ42 (IQR), pg/mL	1147 (809, 1601)	1330 (955, 1633)	711 (618, 923)	<.0001
Median CSF Aβ42/Aβ40 (IQR) ratio	0.099 (0.72, 0.107)	0.102 (0.094, 0.108)	0.050 (0.040, 0.062)	<.0001
Median CSF t‐tau (IQR), pg/mL	356 (311, 444)	349 (311, 416)	444 (325, 542)	.069
Median CSF p‐tau181 (IQR), pg/mL	47.5 (36.8, 57.8)	44.9 (34.0, 53.4)	66.9 (54.5, 86.0)	<.0001
Median CSF Aβ42/t‐tau ratio (IQR)	3.30 (2.10. 4.41)	4.11 (2.85, 4.58)	1.83 (1.14, 2.04)	<.0001
Median CSF Aβ42/p‐tau181 ratio (IQR)	28.8 (14.7, 34.5)	30.9 (26.9, 36.4)	11.1 (6.9, 13.4)	<.0001
Mesoscale discovery platform results				
Median CSF Aβ38 (IQR), pg/mL	3171 (2554, 3778)	3104 (2503, 3780)	3392 (2914, 3753)	.308
Median CSF Aβ40 (IQR), pg/mL	7066 (6254, 8338)	7052 (6151, 8330)	7452 (6562, 8588)	.486
Median CSF Aβ42 (IQR), pg/mL	739 (514, 857)	801 (607, 942)	471 (350, 528)	.0001
Median CSF Aβ42/Aβ40 (IQR) ratio	0.108 (0.081, 0.117)	0.113 (0.105, 0.118)	0.058 (0.047, 0.073)	<.0001
INNOTEST platform results				
Median CSF Aβ42 (IQR), pg/mL	1111 (815, 1406)	1252 (1006, 1458)	669 (560, 788)	<.0001
Median CSF t‐tau (IQR), pg/mL	372 (277, 436)	355 (250, 431)	477 (382, 585)	.0003
Median CSF p‐tau181 (IQR), pg/mL	57.3 (43.3, 70.8)	52.6 (37.4, 66.8)	79.5 (56.3, 88.4)	.001
Median CSF Aβ42/t‐tau ratio (IQR)	3.56 (2.32, 4.57)	3.86 (3.22, 4.79)	1.49 (0.93, 1.61)	<.0001
Median CSF Aβ42/p‐tau181 ratio (IQR)	21.4 (15.2, 27.6)	24.4 (20.0, 28.5)	8.6 (7.0, 12.0)	<.0001

Notes: *P* values are from *t* tests for normally distributed variables (age), χ2 tests of proportion for binary variables (sex and *APOE* ε4 carrier status), and Wilcoxon rank‐sum tests for skewed continuous variables. CSF Aβ42 measurements on the Lumipulse platforms (and their associated ratios) were retrospectively standardized to the CSF Aβ42 certified reference material.^29^

Abbreviations: Aβ, amyloid beta; *APOE*, apolipoprotein E gene; CSF, cerebrospinal fluid; IQR, interquartile ratio; LP, lumbar puncture; MMSE, Mini‐Mental State Examination; PET, positron emission tomography; p‐tau181, phosphorylated tau at site 181; SD, standard deviation; t‐tau, total tau.

Differences between amyloid PET groups in CSF t‐tau measured by Lumipulse did not achieve statistical significance (median concentration in pg/mL [IQR]: PET–positive 444 [325, 542] vs PET–negative 349 [311, 416], *P* = .069) but the INNOTEST assay did detect significantly higher CSF t‐tau in the PET–positive group (477 [382, 585] vs 355 [350, 431], *P* = .0003). Both platforms detected significantly higher p‐tau181 in the PET–positive group (median concentration in pg/mL [IQR]: Lumipulse PET–positive 66.9 [54.5, 86.0] vs PET–negative 44.9 [34.0, 53.4], *P* < .0001; INNOTEST 79.5 [56.3, 88.4] vs 52.6 [37.4, 66.8], *P* = .001).

The Aβ42/t‐tau ratio was significantly lower in PET–positive individuals (median ratio [IQR]: Lumipulse PET–positive 1.83 [1.14, 2.04] vs negative 4.11 [2.85, 4.58], *P* < .0001; INNOTEST 1.49 [0.93, 1.61] vs 3.86 [3.22, 4.79], *P* < .0001). PET–positive individuals also had significantly lower Aβ42/p‐tau181 ratios (median ratio [IQR]: Lumipulse PET–positive 11.1 ([6.9, 13.4] vs PET–negative 30.9 ([26.9, 36.4], *P* < .0001; INNOTEST 8.6 [7.0, 12.0] vs 24.4 [20.0, 28.5], *P* < .0001).

### 3.2 Correlations between CSF biomarker measurements across platforms

**FIGURE 1:**
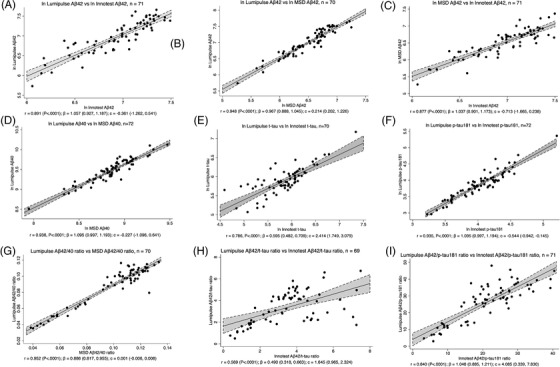
Correlations between measurements of the same biomarkers on different platforms. Individual biomarkers were natural log‐transformed before assessing Pearson correlations and performing linear regression. Ratios of biomarkers were not transformed. The Pearson correlation coefficient r and its P value are shown. The linear regression coefficient 𝛽 and the intercept of the regression line c are shown with their 95% confidence intervals in brackets.

CSF Aβ42 measurements were highly correlated across the three platforms (Lumipulse vs INNOTEST r = 0.891; Lumipulse vs MSD r = 0.948; MSD vs INNOTEST r = 0.887; all *P* < .0001; Figure 1A‐C). CSF Aβ40 was also highly correlated across the Lumipulse and MSD platforms (r = 0.936, *P* < .0001; Figure 1D). Measurements on the Lumipulse and INNOTEST platforms of p‐tau181 were better correlated than those of t‐tau (p‐tau181 r = 0.935, t‐tau 0.786, both *P* < .0001; Figure 1E and F). This was also reflected in correlations of ratios between biomarkers (Figure 1G–I); only a modest correlation was observed for Lumipulse versus INNOTEST Aβ42/t‐tau (r = 0.569, *P* < .0001) but correlations were stronger for Lumipulse versus INNOTEST Aβ42/p‐tau181 (r = 0.840, *P* < .0001) and Lumipulse versus MSD Aβ42/Aβ40 (r = 0.952, *P* < .0001).

### 3.3 Spiked Aβ40 interference

Spiked Aβ40 did not significantly interfere with Aβ42 measurements by the Lumipulse platform (Figure S1A and S1D in supporting information). However, significant negative correlations between measured Aβ42 and spiked Aβ40 were observed for both the MSD and INNOTEST platforms (MSD rho = –0.893, *P* = .007 for the low Aβ42 sample and rho = –0.786, *P* = .036 for the high Aβ42 sample—Figure S1B and S1E; INNOTEST rho = ‐0.964 and *P* = .0005 for both samples—Figure S1C and SF).

### 3.4 Concordance of CSF biomarkers with amyloid PET

The performance of the three platforms in classifying amyloid PET–negative/positive status is shown in Table 3, for those individual biomarkers and ratios that performed better than chance. For CSF Aβ42 and all ratios incorporating it, AUC were 0.89 or above, with no statistically significant differences between methods. However, specificity at the Youden's index was improved by the use of ratios (86% to 94%) compared to using Aβ42 alone (74% to 86%) measured on any platform. P‐tau181 performed better when measured by Lumipulse (AUC Lumipulse 0.879 vs INNOTEST 0.791, De Long test *P* = .024) but t‐tau performed better when measured by Innotest (AUC Lumipulse 0.665 vs INNOTEST 0.825, *P* = .005). Figure S2 in supporting information shows scatter plots of the raw data, demonstrating the superiority of the ratios compared to individual biomarkers.

**TABLE 3:** Comparison of CSF biomarkers for prediction of amyloid PET status

Biomarker	Platform	AUC	95% CI for AUC	Youden index	Cut‐point (pg/mL)	Specificity (%)	Sensitivity (%)
Aβ42	Lumipulse	0.891	0.811–0.970	0.740	974	74	100
	MSD	0.897	0.821–0.973	0.800	586	80	100
	INNOTEST	0.948	0.895–1.000	0.860	936	86	100
t‐tau	Lumipulse	0.665	0.479–0.851	0.358	443	82	54
	INNOTEST	0.825^a^	0.708–0.941	0.572	442	88	69
p‐tau181	Lumipulse	0.879	0.787–0.970	0.660	49	66	100
	INNOTEST	0.791^b^	0.654–0.927	0.458	77	92	54

Biomarker	Platform	AUC	95% CI for AUC	Youden index	Cut‐point (pg/mL)	Specificity (%)	Sensitivity (%)
Aβ42/Aβ40	Lumipulse	0.966	0.922–1.000	0.940	0.075	94	100
	MSD	0.966	0.921–1.000	0.940	0.087	94	100
Aβ42/t‐tau	Lumipulse	0.955	0.906–1.000	0.823	2.17	90	92
	INNOTEST	0.960	0.912–1.000	0.900	2.61	90	100
Aβ42/p‐tau181	Lumipulse	0.966	0.920‐1.000	0.940	17.3	94	100
	INNOTEST	0.934	0.873‐0.995	0.860	17.7	86	100

Notes: The area under the receiver operating characteristics curve (AUC), its 95% confidence interval, the Youden index (at which the combination of sensitivity and specificity is maximized), and the corresponding optimal cut‐point are shown for each of CSF Aβ42, t‐tau, p‐tau181, and their ratios in predicting amyloid PET status (n = 63).

^a^ Higher than AUC for Lumipulse t‐tau, De Long test *P* = .005.

^b^ Lower than AUC for Lumipulse p‐tau181, De Long test *P* = .024.

Abbreviations: Aβ, amyloid beta; AUC, area under the curve; CSF, cerebrospinal fluid; IQR, interquartile ratio; PET, positron emission tomography; p‐tau181, phosphorylated tau at site 181; t‐tau, total tau.

**FIGURE 2:**
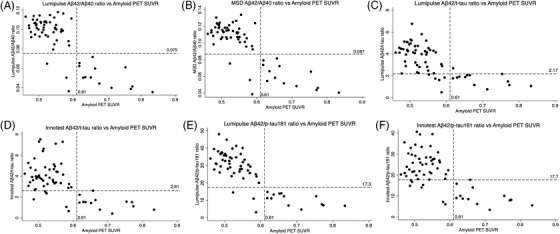
Scatter plots of cerebrospinal fluid (CSF) biomarker ratios (y axis) against standardized uptake value ratio (SUVR; x axis), n=63. Dashed horizontal lines show the Youden’s index cut‐points for the CSF ratios, below which an individual was classified as CSF–positive; dashed vertical lines show the ^18^F‐florbetapir amyloid PET SUVR cut‐point, to the right of which an individual was classified as amyloid PET–positive.

Concordance of CSF biomarker ratios with amyloid PET SUVR as a continuous variable is shown in Figure 2A‐F. The percentage of discordant individuals was low (4% to 11%) and all discordantly classified individuals were CSF–positive and PET–negative, except when the Lumipulse Aβ42/t‐tau ratio was used (one individual was classified as CSF–negative but PET–positive). All discordantly‐classified individuals were male, and 57% to 67% were *APOE* ε4 carriers. Despite this, incorporating age, sex, and *APOE* ε4 carrier status as covariates into predictive models did not significantly change the percentage of discordantly classified individuals or type of discordance (Table S2 and Figure S3 in supporting information).

## 4 DISCUSSION

In this study we build on previous validations of Lumipulse measurements of CSF Aβ and tau biomarkers against two other established CSF assay platforms. We report good correlations of measurements of individual biomarkers of CSF Aβ40, Aβ42, t‐tau, and p‐tau181 between platforms, in agreement with other studies.^18^, ^19^, ^20^ We found a stronger correlation between Lumipulse and MSD measurements of Aβ42/Aβ40 ratio compared to Aβ42 alone. The INNOTEST and MSD platforms showed interference by spiked Aβ40 in measurements of Aβ42, but the Lumipulse platform did not. All ratios incorporating Aβ42 were more concordant with amyloid PET than individual biomarkers; the Lumipulse and MSD Aβ42/Aβ40 and Lumipulse Aβ42/p‐tau181 ratios produced the highest accuracy. The Lumipulse Aβ42/Aβ40 cut‐point of 0.075 pg/mL and Aβ42/p‐tau181 cut‐point of 17.3 pg/mL demonstrated 100% sensitivity and 94% specificity for distinguishing PET–positive from PET–negative individuals.

Measurements of p‐tau181 correlated better than t‐tau between Lumipulse and INNOTEST, and this was also reflected in the Aβ42/p‐tau181 and Aβ42/t‐tau ratios. Our findings for t‐tau contrast with high correlations (r > 0.9) reported in other studies between Lumipulse and INNOTEST measurements^19^, ^31^ but cannot be explained in our study by any differences in pre‐analytical handling, as both t‐tau and p‐tau181 were measured on both platforms from the same aliquot of CSF from each individual. Values of t‐tau at the lower end of the range of samples measured were less well correlated, whereas values of p‐tau181 were very well correlated throughout the range measured (Figure 1E and F). It is unclear whether this reflects altered performance of one or both of the t‐tau assays in this part of the measurement range, but it is important to note that the values were well above the published lower limits of quantification for both assays.

We found differences in absolute biomarker values between platforms, as reported by others.^18^, ^19^ For MSD values this could in part be due to different antibodies used for Aβ measurements; other reasons could include differences in the technology and calibrators used. Of the three Aβ42 assays, only the Lumipulse assay had been retrospectively calibrated against the CSF Aβ42 CRM,^32^ which was developed to provide an international standard for this analyte, and CRMs are not yet available for the other analytes. Furthermore, it is possible that native Aβ40 leads to inaccurate Aβ42 estimates in the INNOTEST assay as previously reported.^13^, ^33^ Lumipulse was the only platform of the three that did not show significant interference of spiked Aβ40 with Aβ42 measurements. We found that both the INNOTEST and MSD platforms showed about 25% reduction of measurements of Aβ42 when spiking up to 40 ng/mL of Aβ40, in contrast to Janelidze et al.,^13^ who showed 60% reduction for INNOTEST and 20% reduction for MSD with similar Aβ40 spiking concentrations. Although the Lumipulse Aβ42 assay uses the same monoclonal antibodies as INNOTEST, it is likely that Lumipulse is less susceptible to matrix effects, based on the optimal minimal required dilution of sample in the conjugate solution. We did find significant Aβ40 interference with MSD measurements; these could be due to similar matrix effects as found on the INNOTEST, or due to differences in antibody specificity between the two assays.

We report 100% sensitivity across all three platforms for CSF Aβ42 in predicting cortical amyloid load, but the specificity of the Lumipulse measurements (74%) was lower than that of the INNOTEST (86%) or MSD (80%). In contrast to other studies, which show CSF t‐tau and p‐tau181 to be good individual predictors of amyloid PET,^24^, ^25^ we found that performance varied by platform; p‐tau181 performed better when measured by Lumipulse compared to INNOTEST and the converse was found for t‐tau. Furthermore, in line with previous studies^13^, ^14^, ^24^, ^25^ and extended to incorporate all platforms assessed, we show that all ratios incorporating Aβ42 improved concordance with amyloid PET. Lumipulse cut‐points of 0.075 for Aβ42/Aβ40 and 17.3 for Aβ42/p‐tau181 both produced a specificity of 94% and sensitivity of 100% for detecting amyloid PET status. Our optimal cut‐points are slightly higher than those derived by Alcolea et al., who examined Lumipulse CSF biomarker concordance with 18F‐florbetapir PET (0.062 for Aβ42/Aβ40 and 0.068 for p‐tau181/Aβ42, which is equivalent to 14.7 for Aβ42/p‐tau181).^24^ Our cut‐point of 2.17 for Lumipulse Aβ42/t‐tau is also slightly higher than the cut‐point of 1.85 (equivalent to 0.54 for Lumipulse t‐tau/Aβ42) derived by Kaplow et al.^25^ Possible explanations for these differences include differing definitions of amyloid PET–positivity (Alcolea et al. used the cerebellum as the SUVR reference region and Kaplow et al. used a variety of PET tracers), a number of individuals in our cohort being close to the SUVR cut‐point, and participants in the other studies having a wider range of cognitive performance and overall higher prevalence of *APOE* ε4 carriage than in the participants of our cohort.

Advantages of the Lumipulse platform over conventional assays include reduction in labor‐intensive steps and manual error; reduced total analysis time due to testing all four biomarkers on the same CSF sample; and improved accuracy for analyte detection, due to the measurement by photon‐counting of directly emitted light rather than wavelength‐based colorimetric absorbance. Furthermore, the Lumipulse platform can process small numbers of CSF samples, without needing to collect enough samples to use in batched assays (as is required for the INNOTEST or MSD). However, a disadvantage of the Lumipulse is its requirement for a large dead volume of 100 µL, relative to sample volumes per replicate for the four biomarkers of 40 to 75 µL, while the INNOTEST and MSD assays use similar volumes per replicate (25 to 75 µL) but have no dead volume requirement.

This study has some limitations that might be assessed in future research. MSD analysis took place on a separate day using a separate sample aliquot to that used for INNOTEST and Lumipulse analysis. Lumipulse measurements were performed in singleton due to CSF volume requirements, so precision of the Lumipulse assays was not assessed. We did not compare measurements of all four biomarkers on all platforms, and we focused on comparing Lumipulse measurement with other immunoassays but not with other methods of measurement like mass spectrometry. Although the AUC obtained for prediction of amyloid PET status were higher for CSF Aβ42 and its ratios than that obtained by the model using age, sex, and *APOE* ε4 carrier status, the differences did not reach statistical significance, likely due to this being an interim dataset of samples collected by this point of the ongoing study. This cohort consists mostly of cognitively healthy individuals of the same age. It is possible that some individuals classified as “CSF–positive” (through the use of the ratio cut‐points) but “PET–negative” do actually have subthreshold cerebral amyloid deposition, as CSF changes may precede PET changes.^34^ However, in the absence of neuropathological data to date in this cohort, the use of amyloid PET as an in vivo “gold standard” is a necessary limitation.

In summary, this study supports the use of the fully automated Lumipulse platform, particularly for measuring CSF Aβ42/Aβ40 and Aβ42/p‐tau181, to identify cerebral amyloid deposition with excellent sensitivity and high specificity, without Aβ40 interference, even in cognitively normal individuals.

## CONFLICTS OF INTEREST

Avid Radiopharmaceuticals, a wholly owned subsidiary of Eli Lilly, kindly provided the 18F‐florbetapir tracer (Amyvid) free of cost but had no role in the design, conduct, analysis, or reporting of Insight 46 study findings. We are particularly indebted to the support of the late Chris Clark of Avid Radiopharmaceuticals who championed this study from its outset. Fujirebio provided and set up the Lumipulse platform free of cost but had no role in the design, conduct, analysis, or reporting of this study. The National Survey of Health and Development is funded by the Medical Research Council (MC_UU_00019/1, MC_UU_00019/3). TDP was supported by a Wellcome Trust Clinical Research Fellowship (200109/Z/15/Z). NCF is supported by UK Dementia Research Institute at University College London, Medical Research Council, National Institute for Health Research (Senior Investigator award), and Engineering and Physical Sciences Research Council. HZ is a Wallenberg Scholar supported by grants from the Swedish Research Council (#2018‐02532), the European Research Council (#681712), Swedish State Support for Clinical Research (#ALFGBG‐720931), the Alzheimer Drug Discovery Foundation (ADDF), USA (#201809‐2016862), and the UK Dementia Research Institute at UCL. HZ has served at scientific advisory boards for Denali, Roche Diagnostics, Wave, Samumed, and CogRx; has given lectures in symposia sponsored by Fujirebio, Alzecure, and Biogen; and is a co‐founder of Brain Biomarker Solutions in Gothenburg AB (BBS), which is a part of the GU Ventures Incubator Program (all outside the submitted work). JMS is supported by Engineering and Physical Sciences Research Council (EP/J020990/1), British Heart Foundation (PG/17/90/33415), and EU's Horizon 2020 research and innovation programme (666992). MH and JMS are supported by the University College London Hospitals Biomedical Research Centre. NCF and JMS are supported by the National Institute for Health Research Queen Square Dementia Biomedical Research Unit and the Leonard Wolfson Experimental Neurology Centre.

## Supporting information

Supporting Information
