# Supplementary material for: Concordance of CSF measures of Alzheimer's pathology with amyloid PET status in a preclinical cohort: A comparison of Lumipulse and established immunoassays
Source: Alzheimers Dement (Amst). 2021 Feb 6;13(1):e12131. doi: 10.1002/dad2.12131 (PMC7867115; doi:10.1002/dad2.12131)
Supplement: Supplementary file 1 — Supporting Information [file DAD2-13-e12131-s001.docx]

**Concordance of CSF measures of Alzheimer’s pathology with amyloid PET status in a preclinical cohort: a comparison of Lumipulse and established immunoassays**

Keshavan *et al.*

**Supplementary online content**

Supplementary Methods

*A*$\beta$*40 interference*

To test for the effect of A$\beta$40 interference on measurement of A$\beta$42, recombinant A$\beta1-$40 (Ultrapure, TFA, rPeptide, Bogart) was spiked into two CSF samples with known low and high concentrations of native A$\beta$42. The “low CSF A$\beta$42” sample had an A$\beta$42 concentration of 240 pg/ml and the “high CSF A$\beta$42” sample had an A$\beta$42 concentration of 950 pg/ml as quantified using the INNOTEST® $\beta$-amyloid 1-42 (Fujirebio) assay. Samples underwent two freeze-thaw cycles before being used for this experiment. Spiking solutions were made by suspending the A$\beta1-$40 peptide in 1% NH_4_OH and sonicating before diluting with the diluent provided in each assay kit. To minimize differences in matrix effects, the volume of spiking solution was fixed as 10% of the volume of the final sample. Final spiking concentrations of A$\beta$40 were 10, 20, 30, 40, 50 and 60 ng/ml. Each spiked sample was assayed in duplicate on each of the three platforms to measure A$\beta$42 (Lumipulse, MSD and INNOTEST).

| Platform | INNOTEST | | | MSD | |
| --- | --- | --- | --- | --- | --- |
| Biomarker | A$\beta$42 | t-tau | p-tau181 | A$\beta$40 | A$\beta$42 |
| Low run validation control intra-run CV %  Plate 1  Plate 2 | 2.5  10.3 | 3.9  27.8 | 0.2  1.6 | 5.4  0.2 | 2.3  1.9 |
| High run validation control intra-run CV %  Plate 1  Plate 2 | 2.2  6.5 | 1.0  9.0 | 9.2  1.8 | 5.4  5.7 | 7.7  4.8 |
| Low CSF control inter-run CV % | 15.1 | 17.9 | 7.7 | 3.1 | 8.1 |
| High CSF control inter-run CV % | 7.4 | 5.2 | 7.9 | 3.9 | 4.7 |
| Sample CV range % (n) | 0.08 - 26.3  (72) | 0 – 14.6  (70) | 0.01 – 26.8  (72) | 0.01 – 24.3  (72) | 0.04 – 22.9  (71) |

Supplementary table 1: Quantification variation for the INNOTEST and MSD assays. The sample CV range was capped at 30% and n reflects those remaining after exclusion of samples that had a CV above this.

Abbreviations: CV, coefficient of variation

Supplementary table 2: Comparison of characteristics of individuals included vs excluded from this study

|  | All included in PET analysis  n=63 unless otherwise stated | Individuals excluded due to missing PET or CSF data  n=9 unless otherwise stated | *P* | All individuals with available data | |
| --- | --- | --- | --- | --- | --- |
|  |  |  |  |  | n |
| *Demographics* |  |  |  |  |  |
| Mean age at CSF sampling (SD), years | 72.7 (1.3) | 72.3 (0.2) | 0.357 | 72.6 (1.2) | 72 |
| Sex, % male | 71.4 | 55.6 | 0.334 | 69.4 | 72 |
| *APOE* $\varepsilon$4 carrier status, % carrying one or two alleles | 22.6, n=62 | 44.4 | 0.159 | 25.4 | 71 |
| Median MMSE (IQR) | 29 (28, 30) | 30 (28, 30) | 0.260 | 29 (28, 30) | 72 |
| *Lumipulse platform results* |  |  |  |  |  |
| Median CSF A$\beta$40 (IQR), pg/ml | 13193 (10528, 16376) | 12288 (10744, 14025) | 0.568 | 12975 (10586, 16246) | 72 |
| Median CSF A$\beta$42 (IQR), pg/ml | 1147 (809, 1601) | 1020 (696, 1269), n = 8 | 0.267 | 1123 (780, 1511) | 71 |
| Median CSF A$\beta$42/A$\beta$40 (IQR) ratio | 0.099 (0.72, 0.107) | 0.090 (0.056, 0.104), n = 8 | 0.344 | 0.097 (0.067, 0.107) | 71 |
| Median CSF t-tau (IQR) , pg/ml | 356 (311, 444) | 318 (270, 415) | 0.223 | 349 (310, 444) | 72 |
| Median CSF p-tau181 (IQR), pg/ml | 47.5 (36.8, 57.8) | 46.2 (30.9, 67.9) | 0.973 | 47.1 (36.0, 57.9) | 72 |
| Median CSF A$\beta$42/t-tau ratio (IQR) | 3.30 (2.10. 4.41) | 3.03 (1.97, 5.61), n = 8 | 0.957 | 3.30 (2.04, 4.41) | 71 |
| Median CSF A$\beta$42/p-tau181 ratio (IQR) | 28.8 (14.7, 34.5) | 26.8 (12.4, 37.2), n = 8 | 0.716 | 28.3 (14.3, 34.5) | 71 |
| *Mesoscale Discovery Platform results* |  |  |  |  |  |
| Median CSF A$\beta$38 (IQR), pg/ml | 3171 (2554, 3778) | 3236 (2514, 3482) | 0.845 | 3179 (2562, 3694) | 72 |
| Median CSF A$\beta$40 (IQR), pg/ml | 7066 (6254, 8338) | 7076 (5674, 7417) | 0.628 | 7071 (6264, 8309) | 72 |
| Median CSF A$\beta$42 (IQR), pg/ml | 739 (514, 857) | 803 (470, 873), n = 8 | 0.771 | 750 (54, 857) | 71 |
| Median CSF A$\beta$42/A$\beta$40 (IQR) ratio | 0.108 (0.081, 0.117) | 0.115 (0.082, 0.124), n = 8 | 0.383 | 0.110 (0.081, 0.118) | 71 |
| *INNOTEST platform results* |  |  |  |  |  |
| Median CSF A$\beta$42 (IQR), pg/ml | 1111 (815, 1406) | 1090 (811, 1317) | 0.832 | 1100 (815, 1399) | 72 |
| Median CSF t-tau (IQR), pg/ml | 372 (277, 436) | 327 (217, 378), n = 7 | 0.384 | 366 (273, 436) | 70 |
| Median CSF p-tau181 (IQR), pg/ml | 57.3 (43.3, 70.8) | 54.0 (41.3, 71.8) | 0.953 | 56.5 (43.7, 70.6) | 72 |
| Median CSF A$\beta$42/t-tau ratio (IQR) | 3.56 (2.32, 4.57) | 4.15 (1.66, 5.49), n=7 | 0.762 | 3.58 (2.16, 4.59) | 70 |
| Median CSF A$\beta$42 ratio/p-tau181 (IQR) | 21.4 (15.2, 27.6) | 20.8 (12.4, 27.5) | 0.832 | 21.3 (15.4, 27.5) | 72 |

Supplementary table 3: Concordance between CSF and PET biomarkers by incorporation of age, sex and APOE ε4 carrier status into predictive models (n = 62)

|  |  | **Biomarker alone** | | **Biomarker + Age + Sex + *APOE*** $\boldsymbol{\varepsilon}$**4 carrier status** | |
| --- | --- | --- | --- | --- | --- |
| **Biomarker** | **Platform** | **AUC** | **95% CI for AUC** | **AUC** | **95% CI for AUC** |
| **None** | - | - | - | 0.776 | 0.634 – 0.917 |
| **Aβ42** | Lumipulse | 0.889 | 0.807 – 0.970 | 0.920 | 0.850 – 0.990 |
|  | MSD | 0.895 | 0.817 – 0.973 | 0.929 | 0.868 – 0.991 |
|  | INNOTEST | 0.947 | 0.893 – 1.000 | 0.962 | 0.918 – 1.000 |
| **t-tau** | Lumipulse | 0.667 | 0.483 – 0.852 | 0.846 | 0.719 – 0.973 |
|  | INNOTEST | 0.831 | 0.716 – 0.945 | 0.900 | 0.818 – 0.981 |
| **p-tau181** | Lumipulse | 0.885 | 0.797 – 0.974 | 0.926 | 0.855 – 0.998 |
|  | INNOTEST | 0.804 | 0.668 – 0.939 | 0.887 | 0.787 – 0.987 |
| **Aβ42/Aβ40** | Lumipulse | 0.966 | 0.919 – 1.000 | 0.980 | 0.946 – 1.000 |
|  | MSD | 0.966 | 0.910 – 1.000 | 0.977 | 0.945 – 1.000 |
| **Aβ42/t-tau** | Lumipulse | 0.933 | 0.870 – 0.995 | 0.950 | 0.894 – 1.000 |
|  | INNOTEST | 0.959 | 0.910 – 1.000 | 0.978 | 0.946 – 1.000 |
| **Aβ42/p-tau181** | Lumipulse | 0.966 | 0.918 – 1.000 | 0.975 | 0.940 – 1.000 |
|  | INNOTEST | 0.955 | 0.910 – 1.000 | 0.977 | 0.945 – 1.000 |

Supplementary figure 1: Interference of spiked recombinant A$\beta$40 with measurement of A$\beta$42 by the three platforms.

| A | 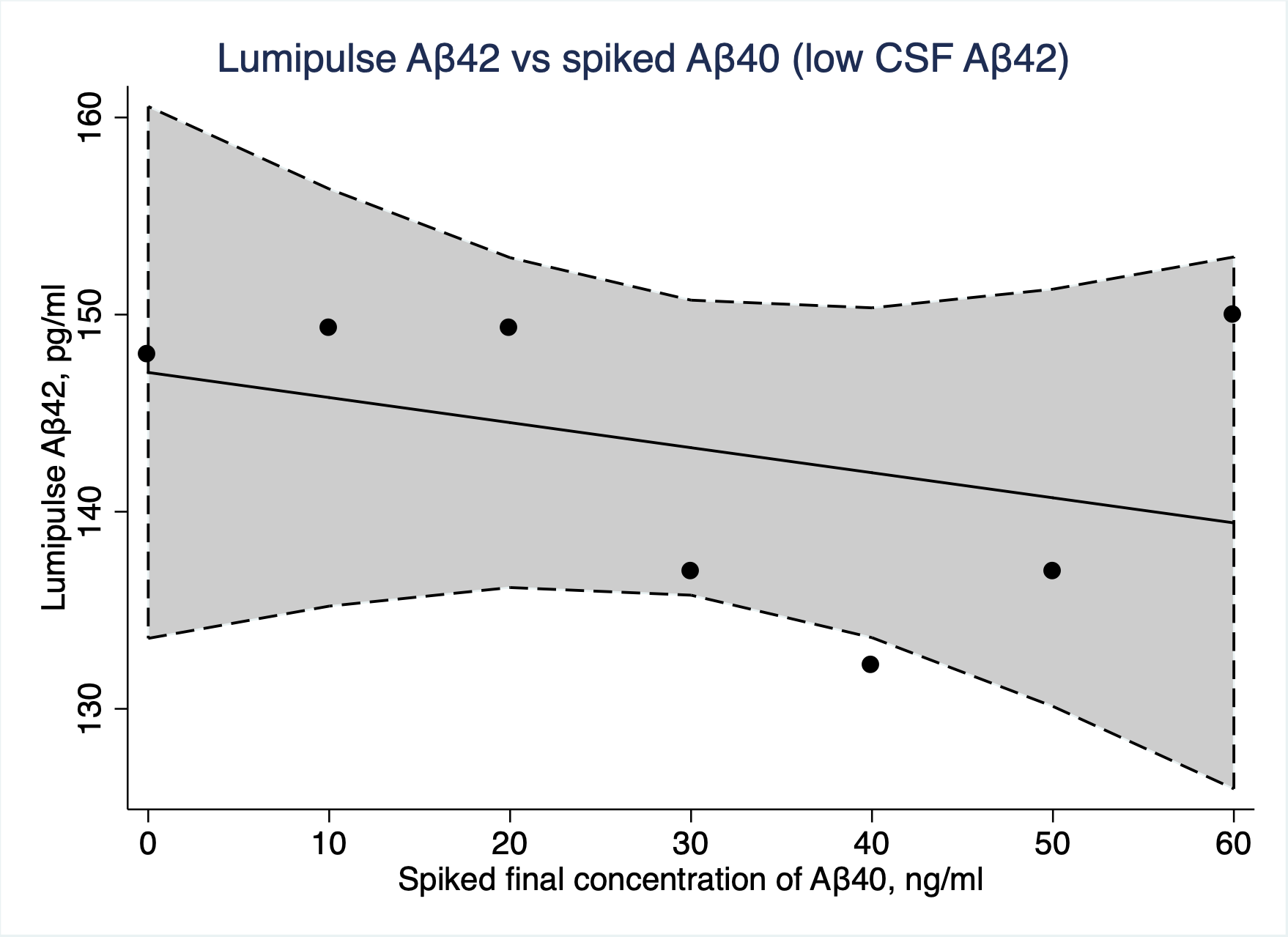  $\beta$ = -0.127 (-0.501, 0.247)  rho = -0.055, *P* = .908 | B | 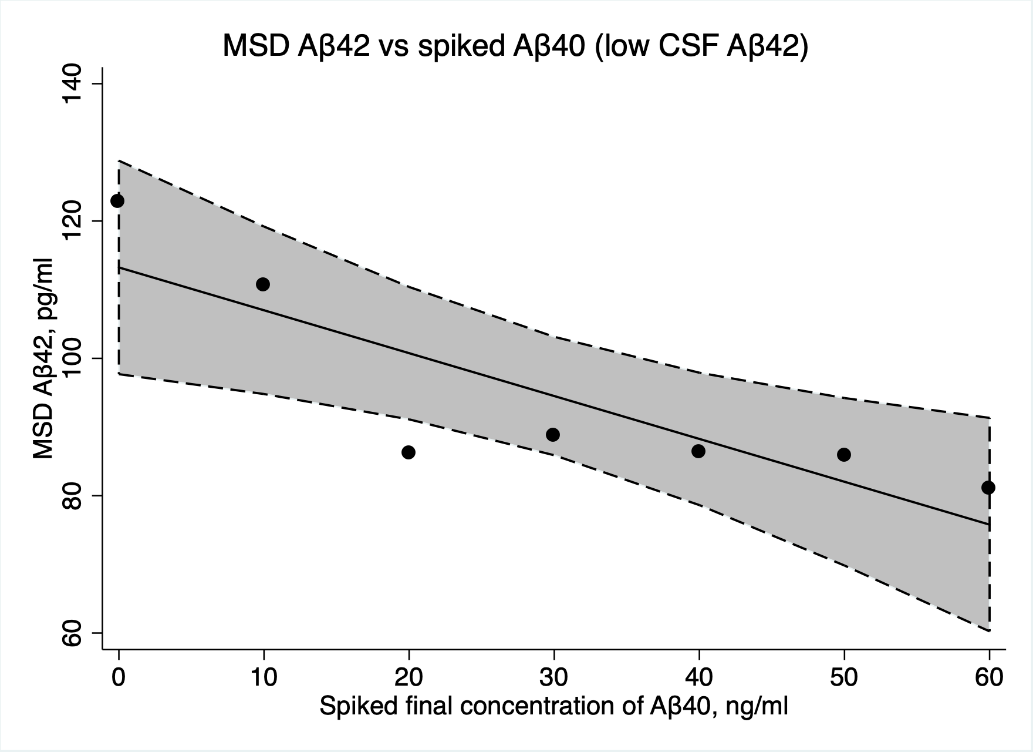  $\beta$ = -0.624 (-1.055, -0.193)  rho = -0.893, *P* = .007 | C | 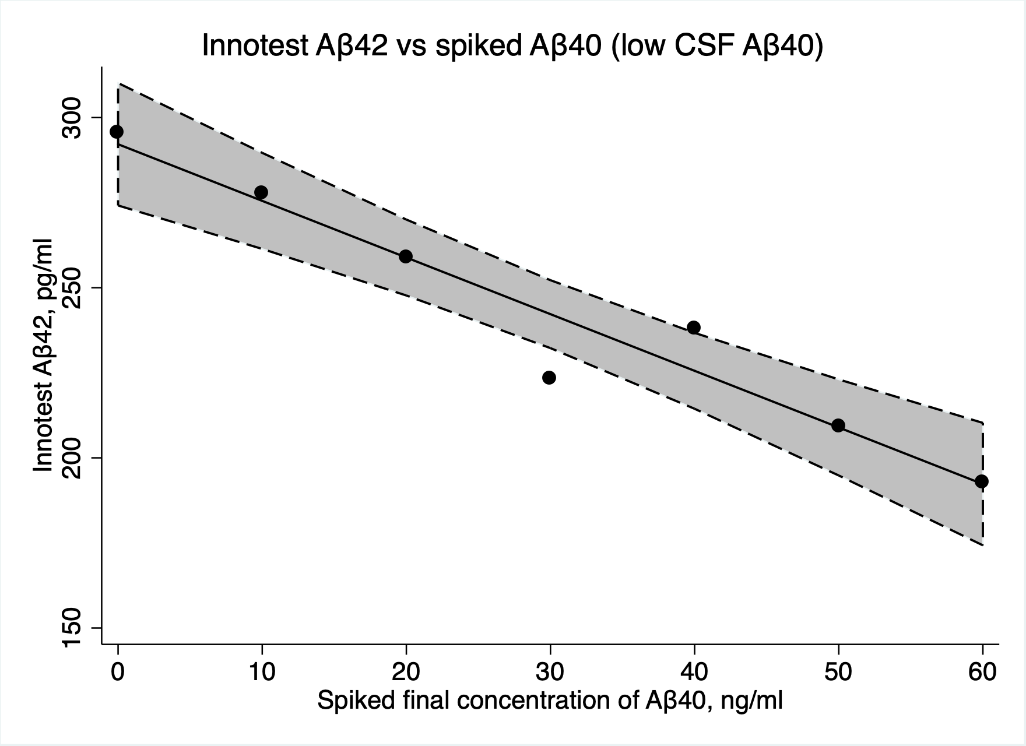  $\beta$ = 1.665 (-2.1634, -1.166), rho = 0.964, *P* = .0005 |
| --- | --- | --- | --- | --- | --- |
| D | 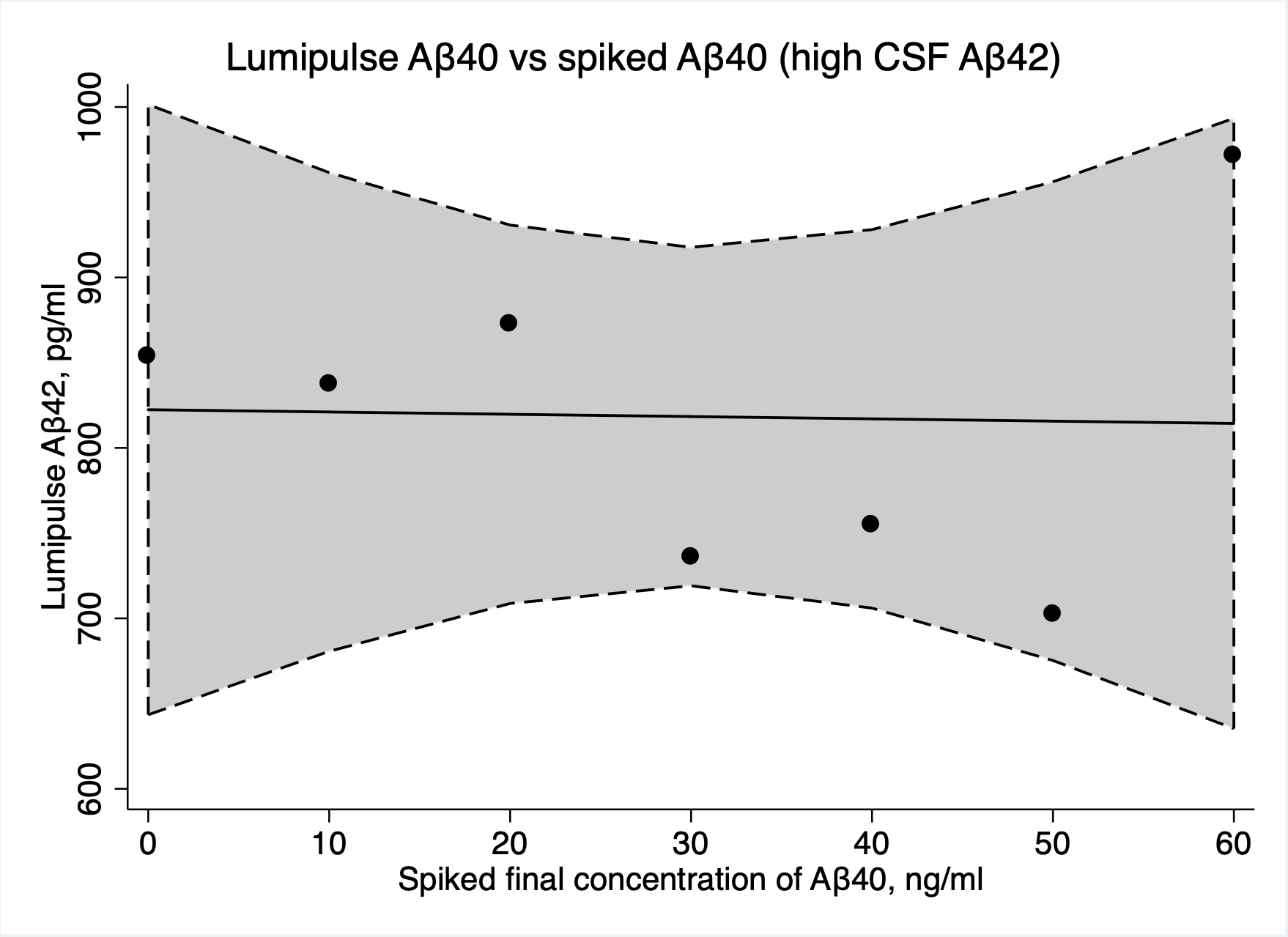  $\beta$ = -0.135 (-5.099, 4.830)  rho = -0.107, *P* = .819 | E | 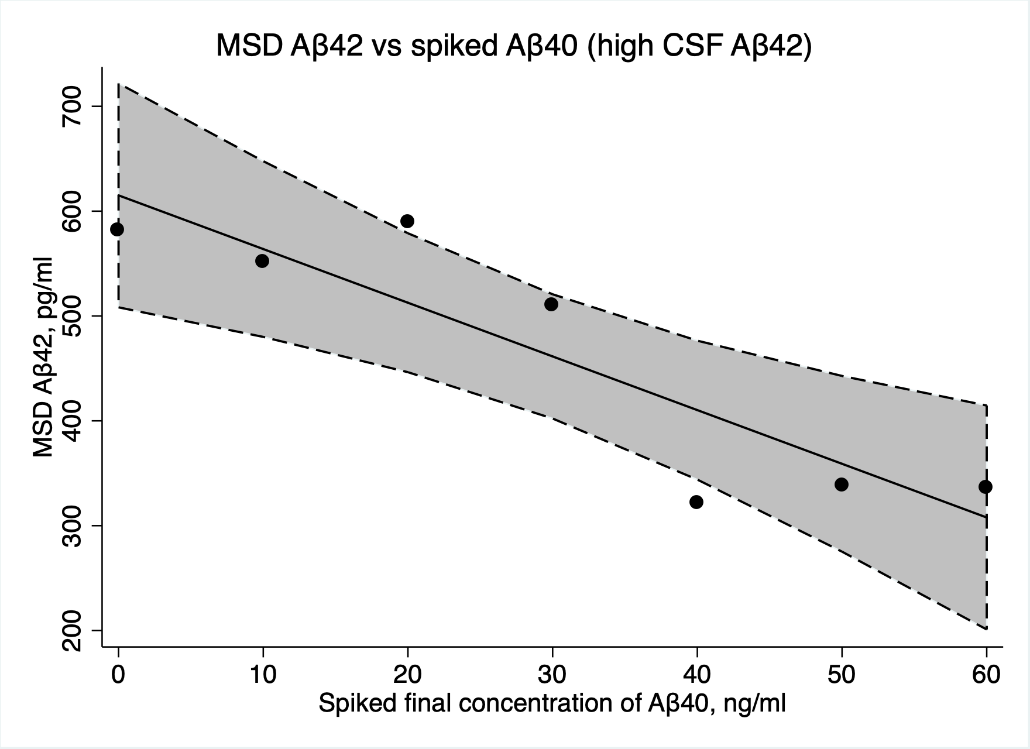  $\beta$ = -5.121 (-8.084, -2.159)  rho = -0.786, *P* = .036 | F | 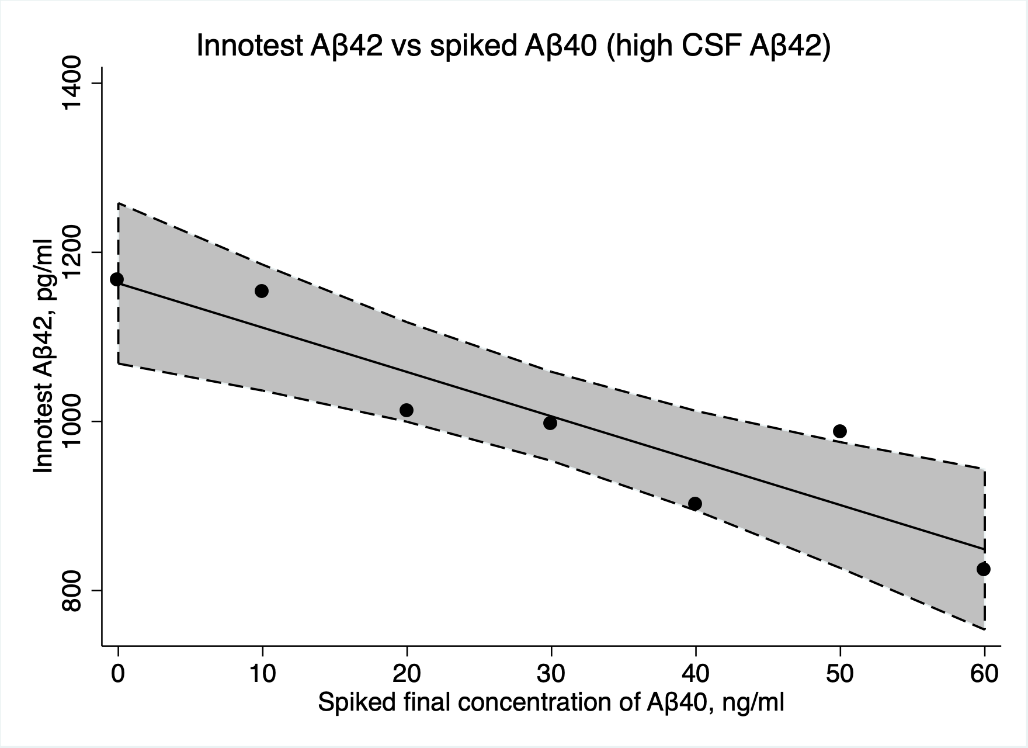  $\beta$ = 5.246 (-7.878, -2.613),  rho = -0.964, *P* = .0005 |
| G | 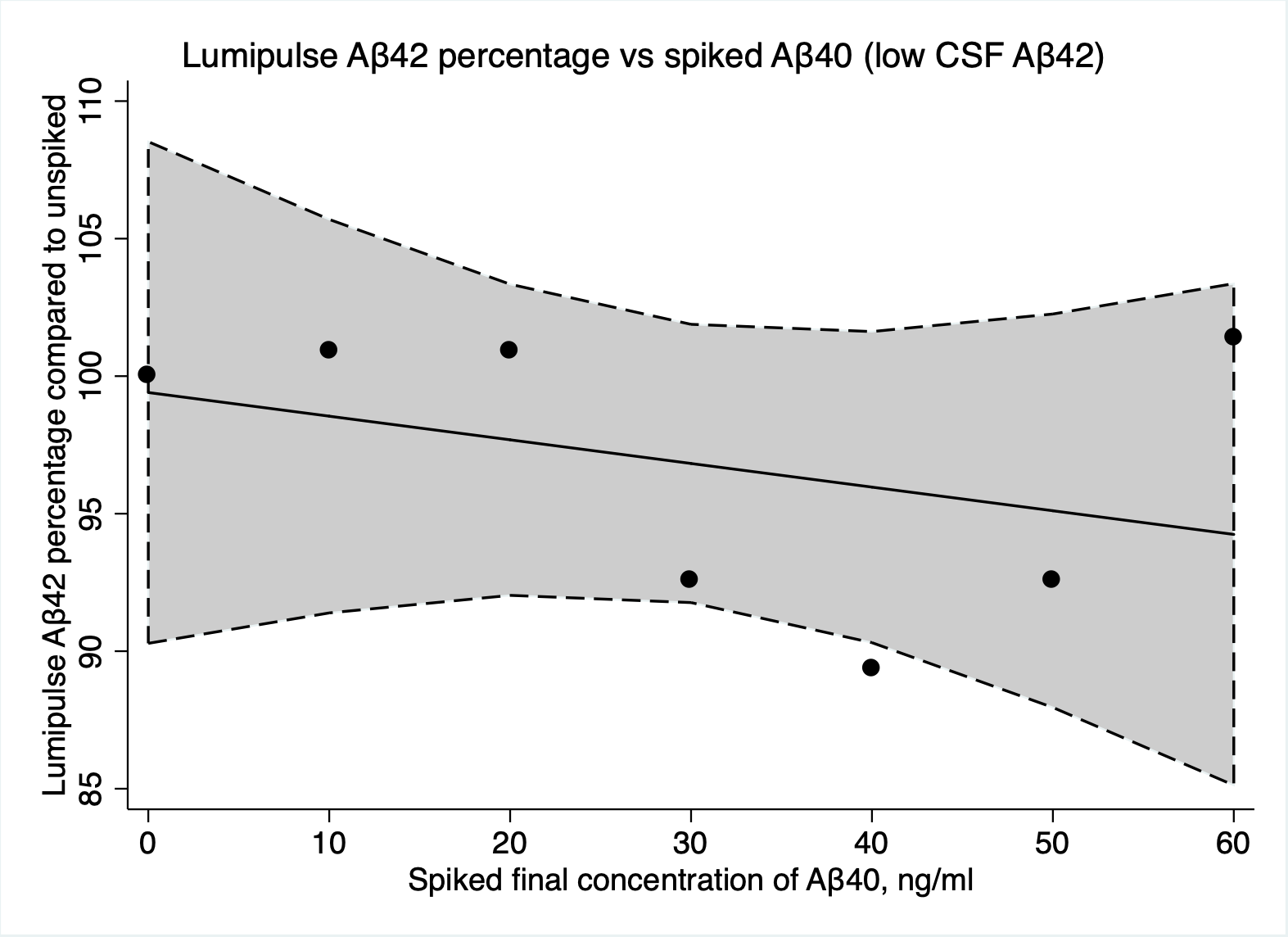  $\beta$ = -0.086 (-0.339, 0.167)  rho = -0.055, *P* = .908 | H | 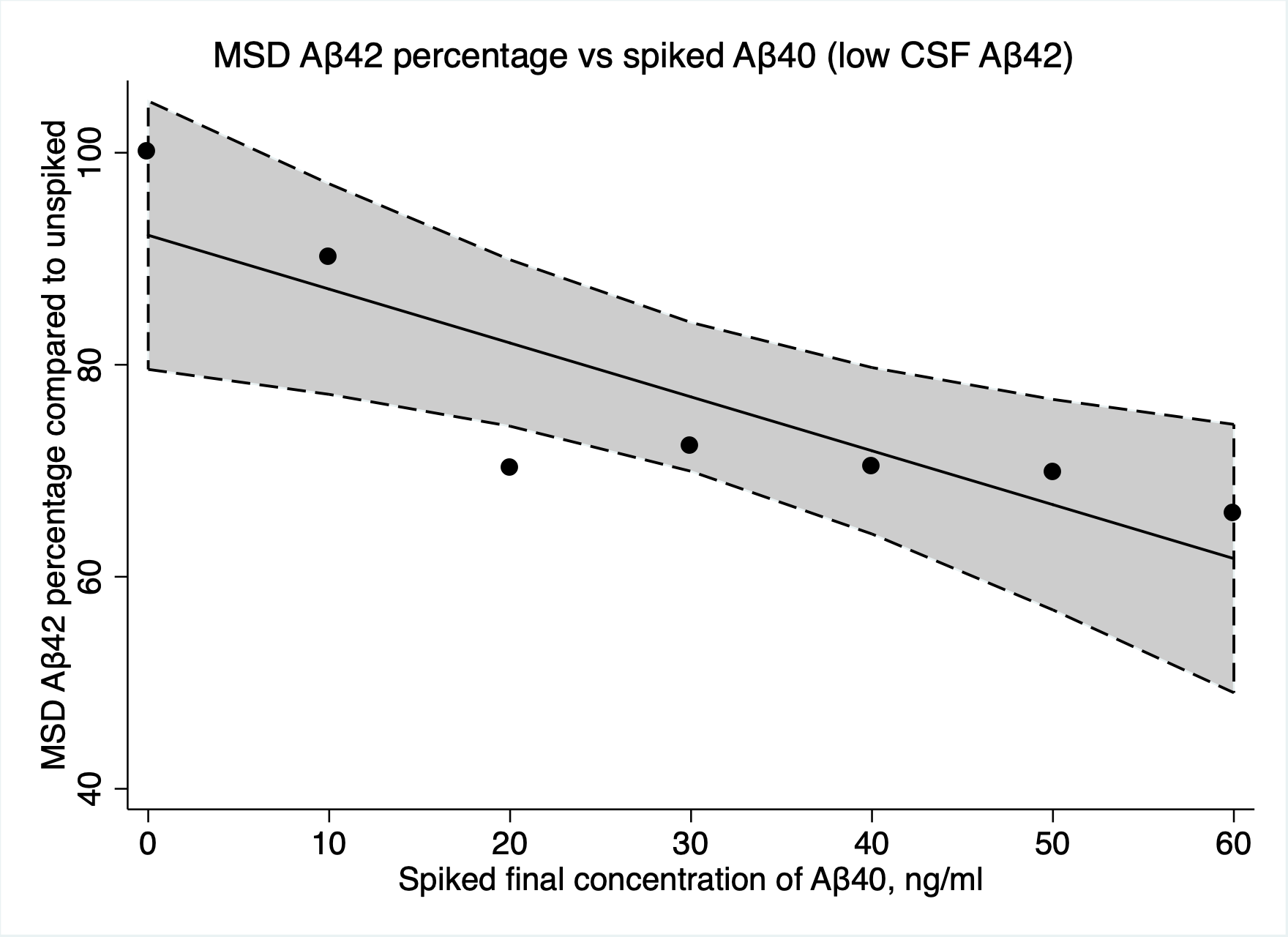  $\beta$ = -0.508 (-0.859, -0.157)  rho = -0.893, *P* = .007 | I | 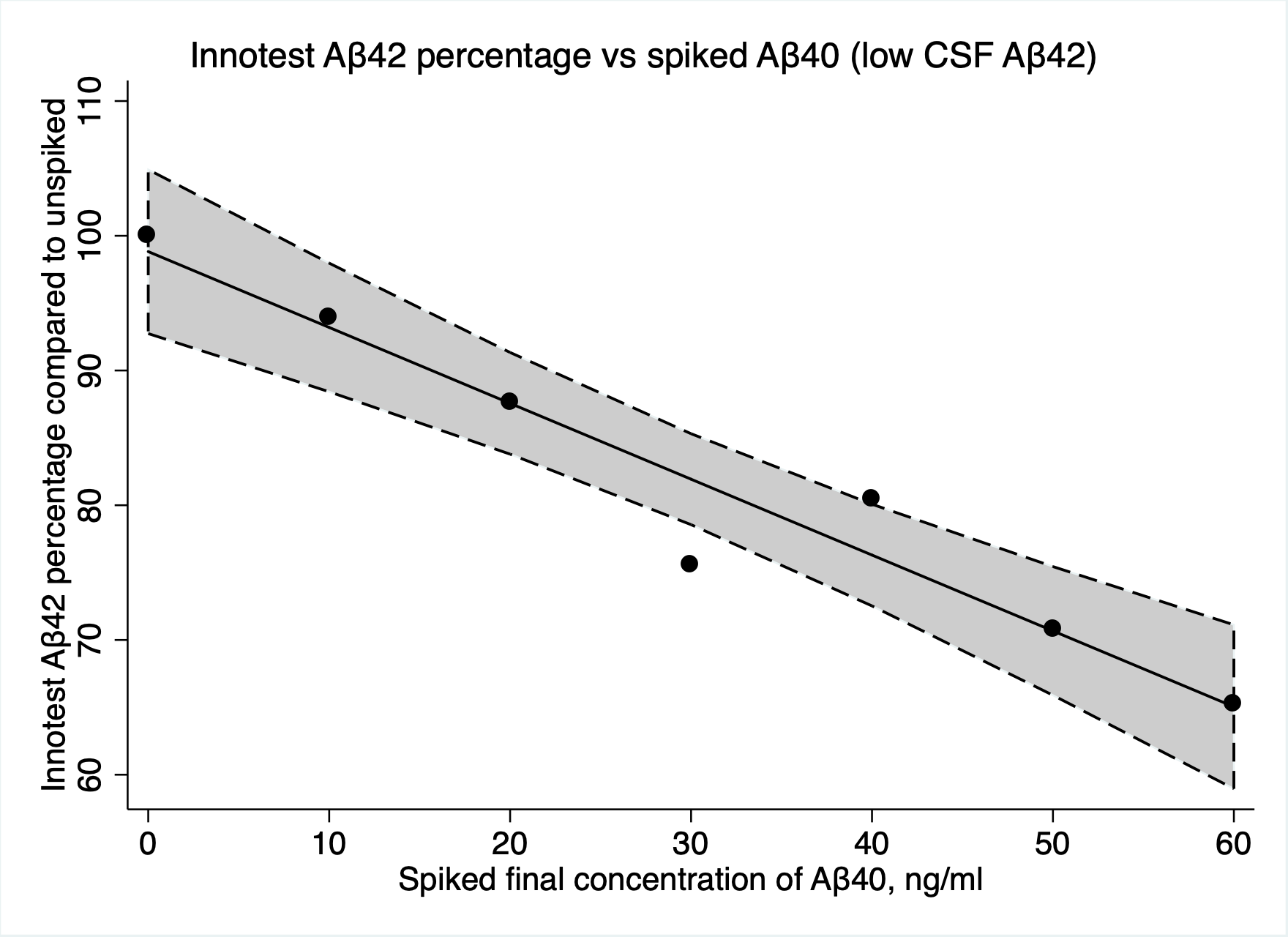  $\beta$ = -0.563 (-0.732, -0.394)  rho = -0.964, *P* = .0005 |
| J | 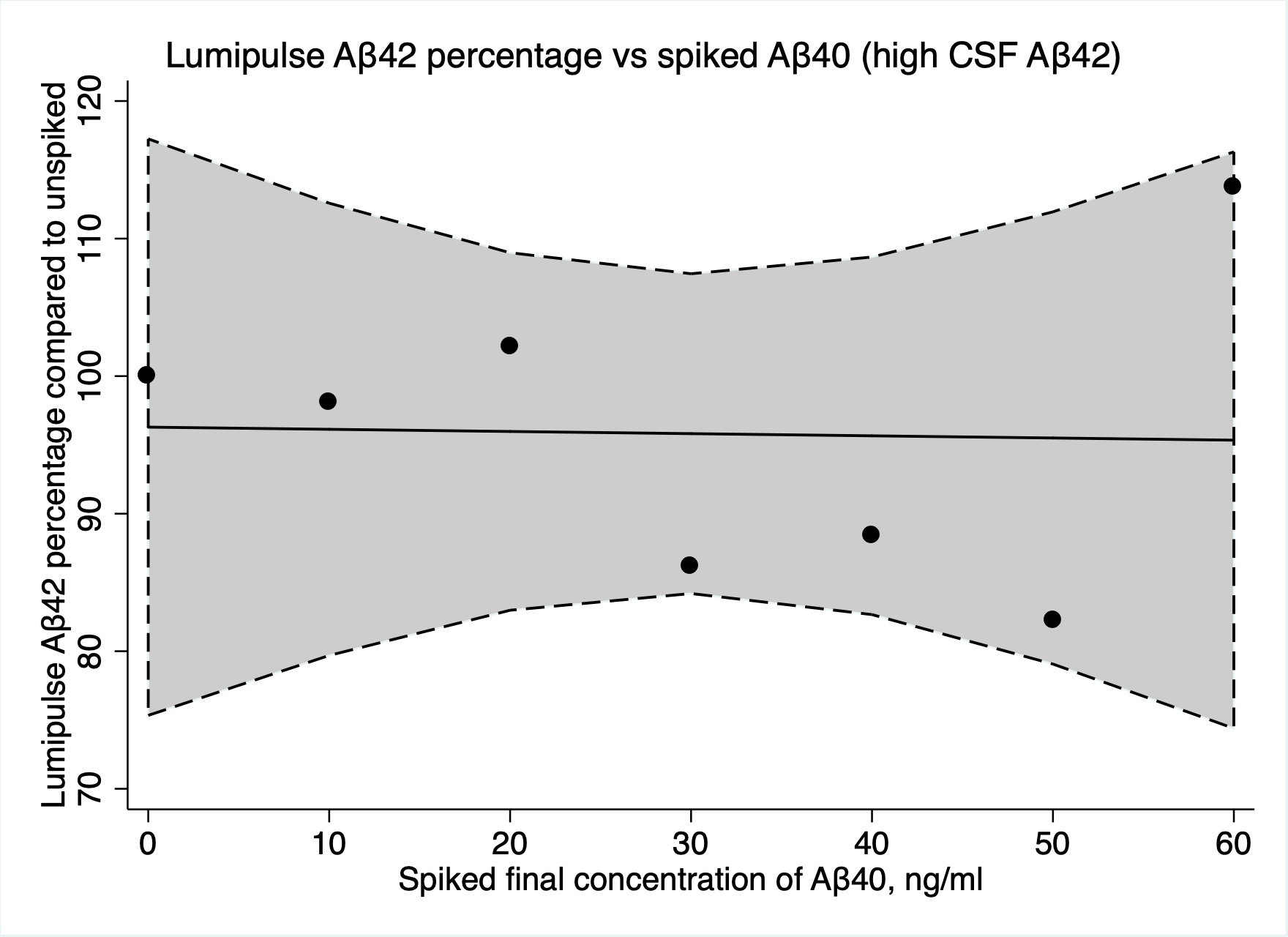  $\beta$ = -0.016 (-0.597, 0.565 )  rho = -0.107, *P* = .819 | K | 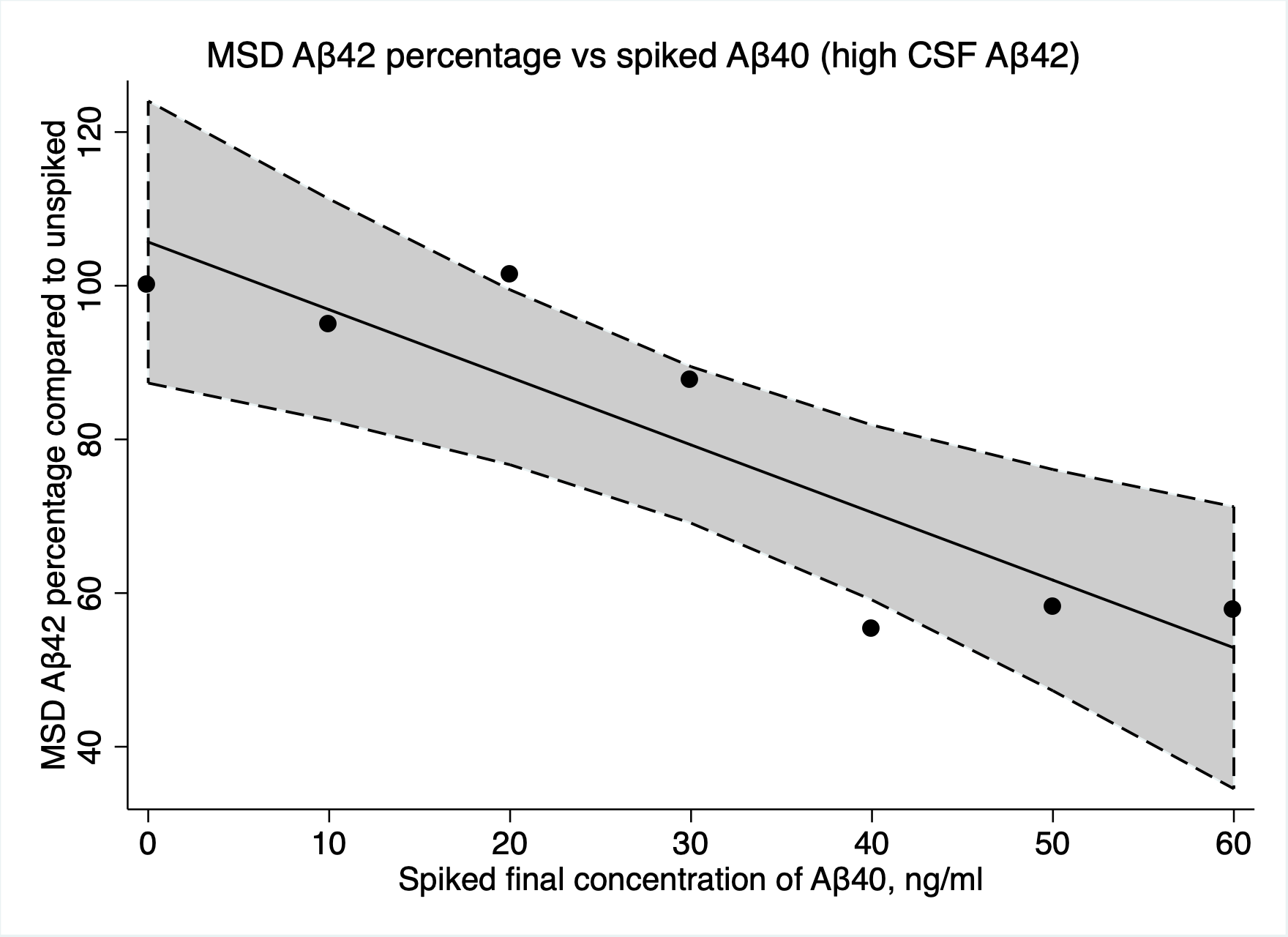  $\beta$ = -0.880 (-1.389, -0.371)  rho = -0.786, *P* = .036 | L | 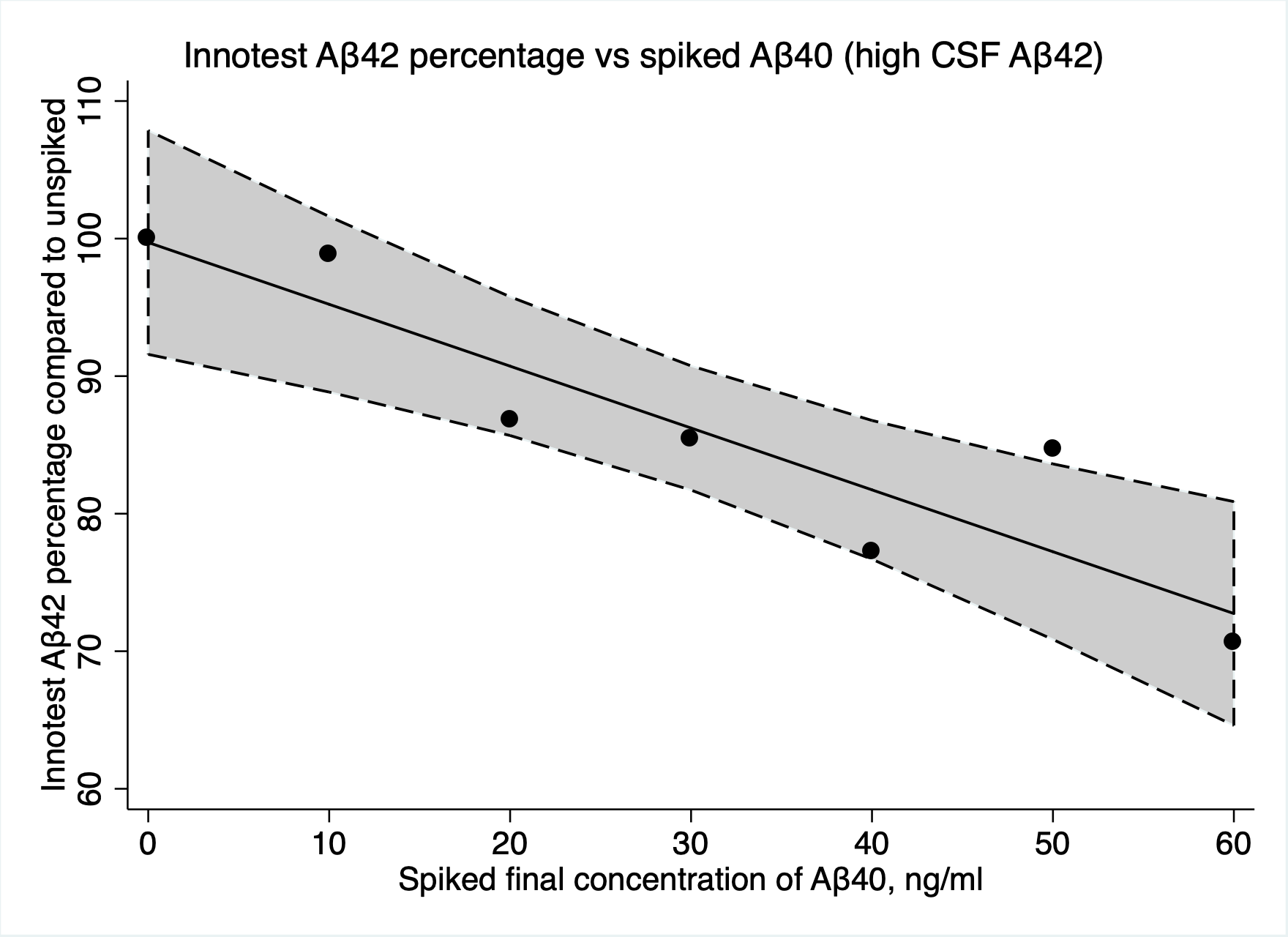  $\beta$ = -0.450 (-0.675, -0.224)  rho = -0.964, *P* = .0005 |

The top row (A, B, C) shows the results of spiking a low CSF A$\beta$42 sample, and the second row (D, E, F) a high CSF A$\beta$42 sample, in terms of absolute A$\beta$42 concentrations. The lower two rows (G, H, I and J, K, L) show the corresponding results in terms of percentage changes in A$\beta$42 concentrations relative to the unspiked sample. Each point is the average of duplicate measurements. The solid line shows the linear correlation between the measured CSF A$\beta$42 value and the known spiked concentration of A$\beta$40, and the grey area its 95% confidence interval. The slope of the line $\beta$ (95% confidence interval), the Spearman correlation coefficient rho and its P value are shown below each graph.

Supplementary figure 2: Use of individual biomarkers vs ratios in separating amyloid PET negative (n=50) and positive (n=13) individuals.

| A | 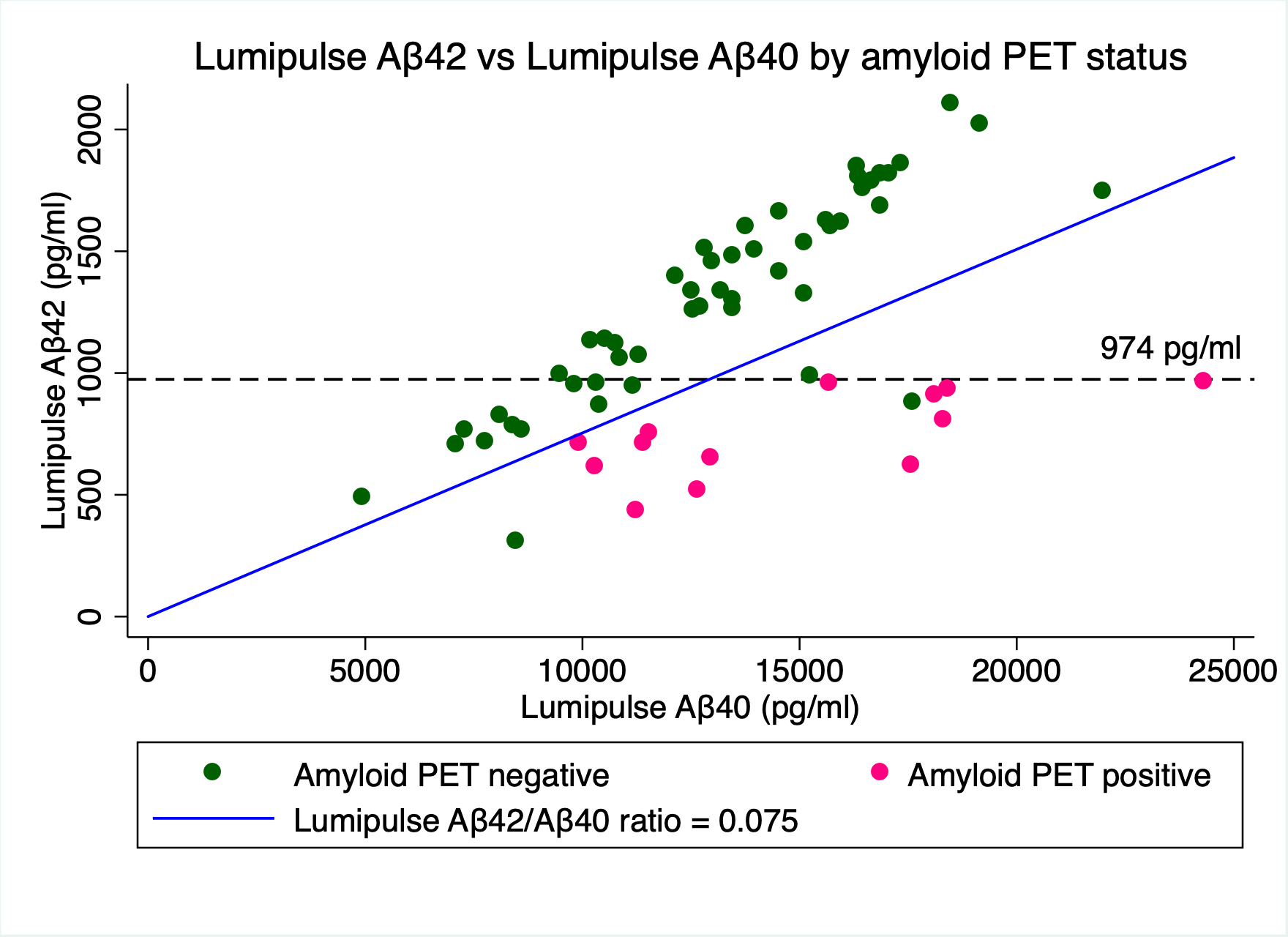 | B | 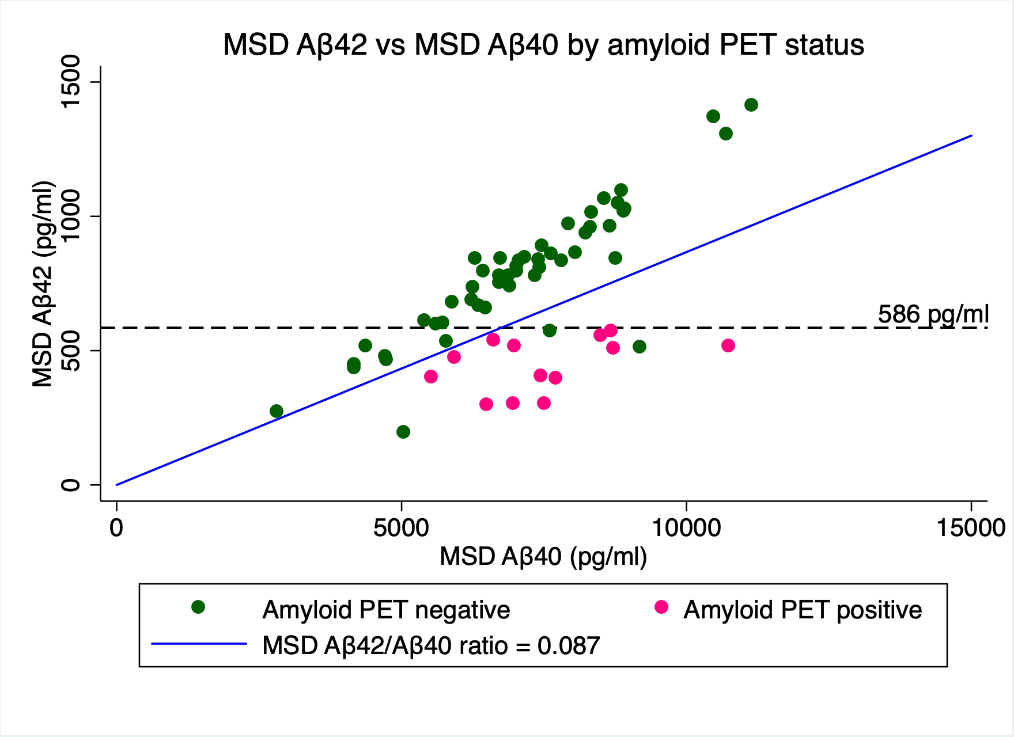 | C | 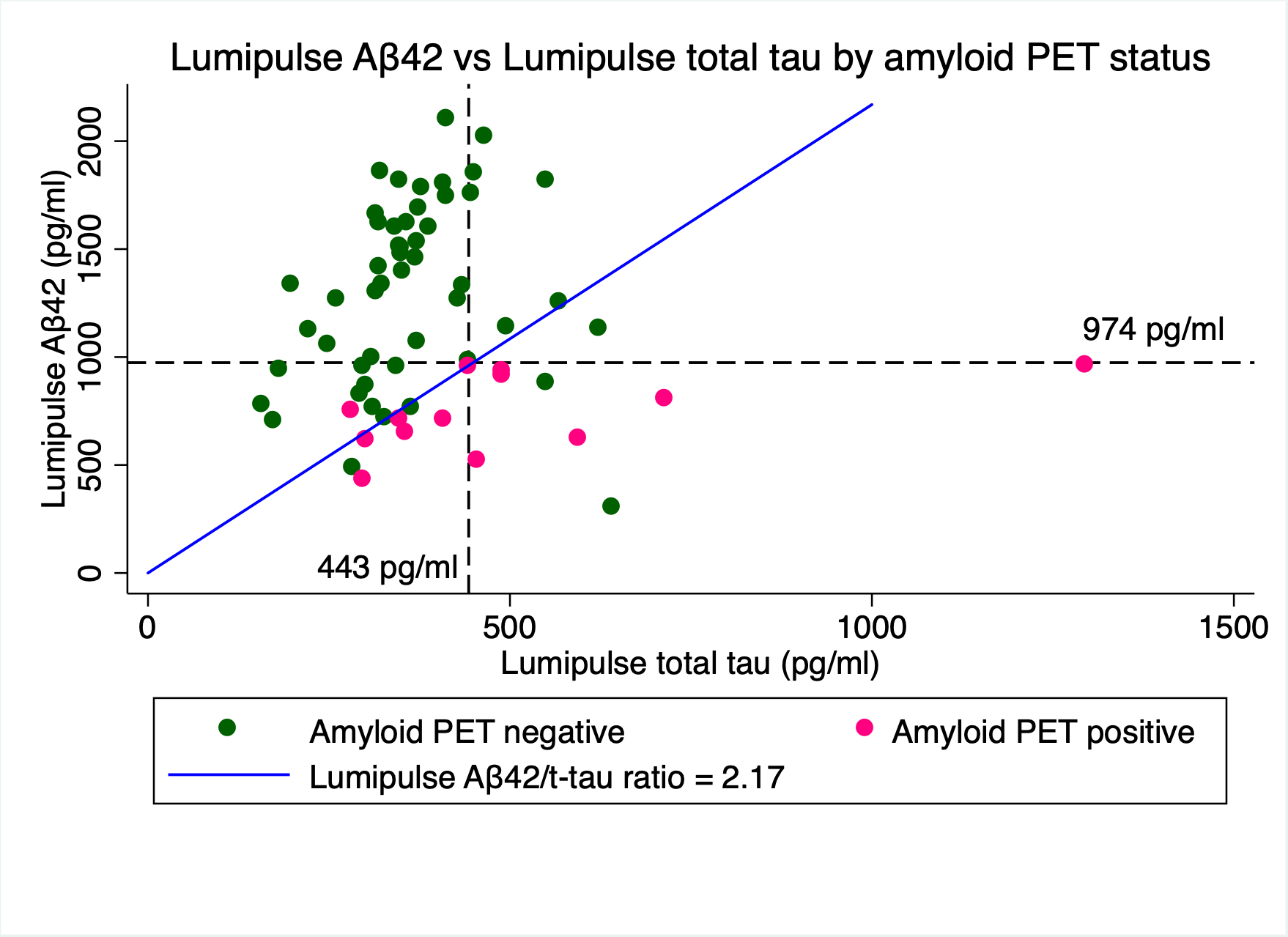 |
| --- | --- | --- | --- | --- | --- |
| D | 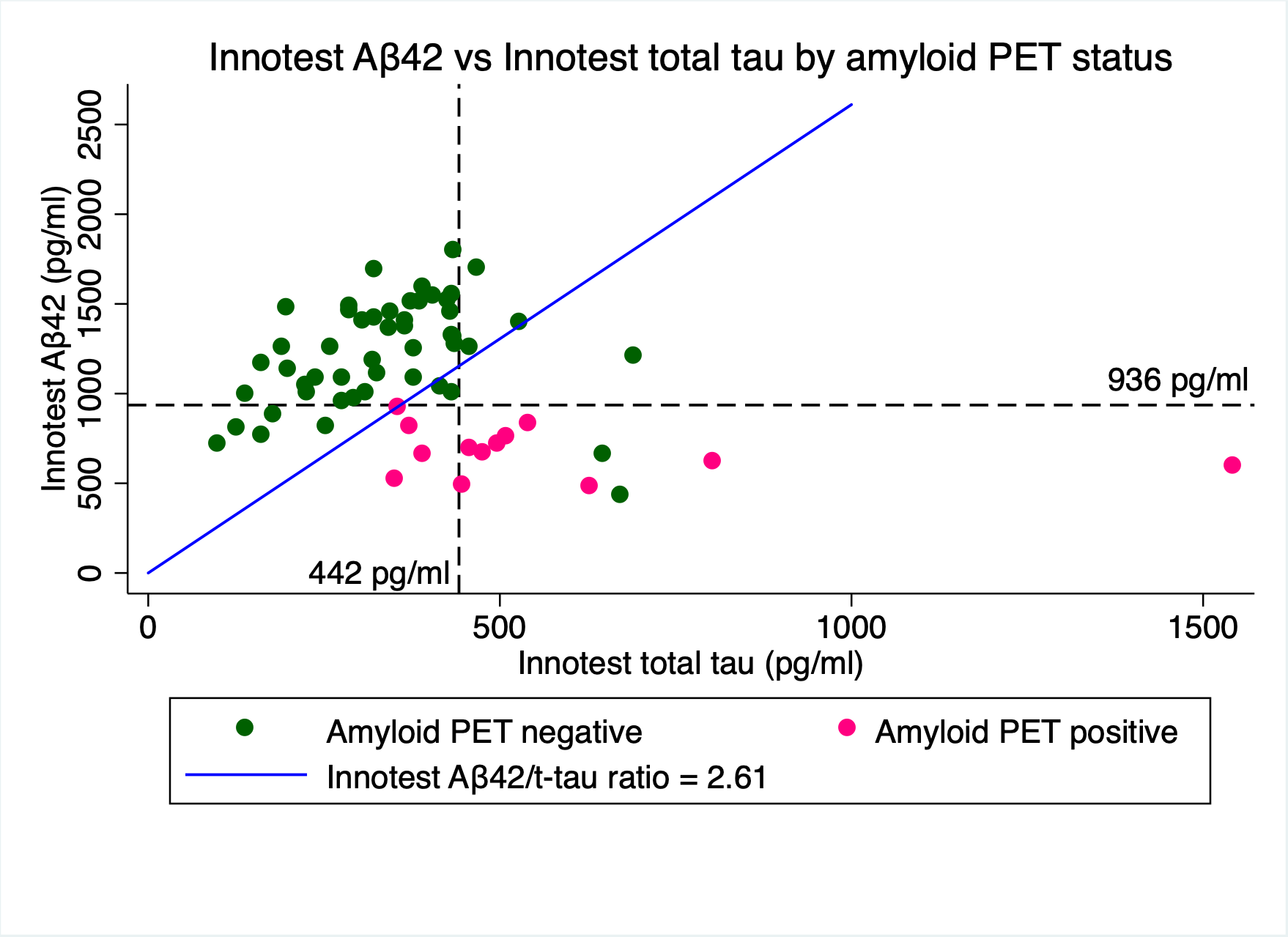 | E | 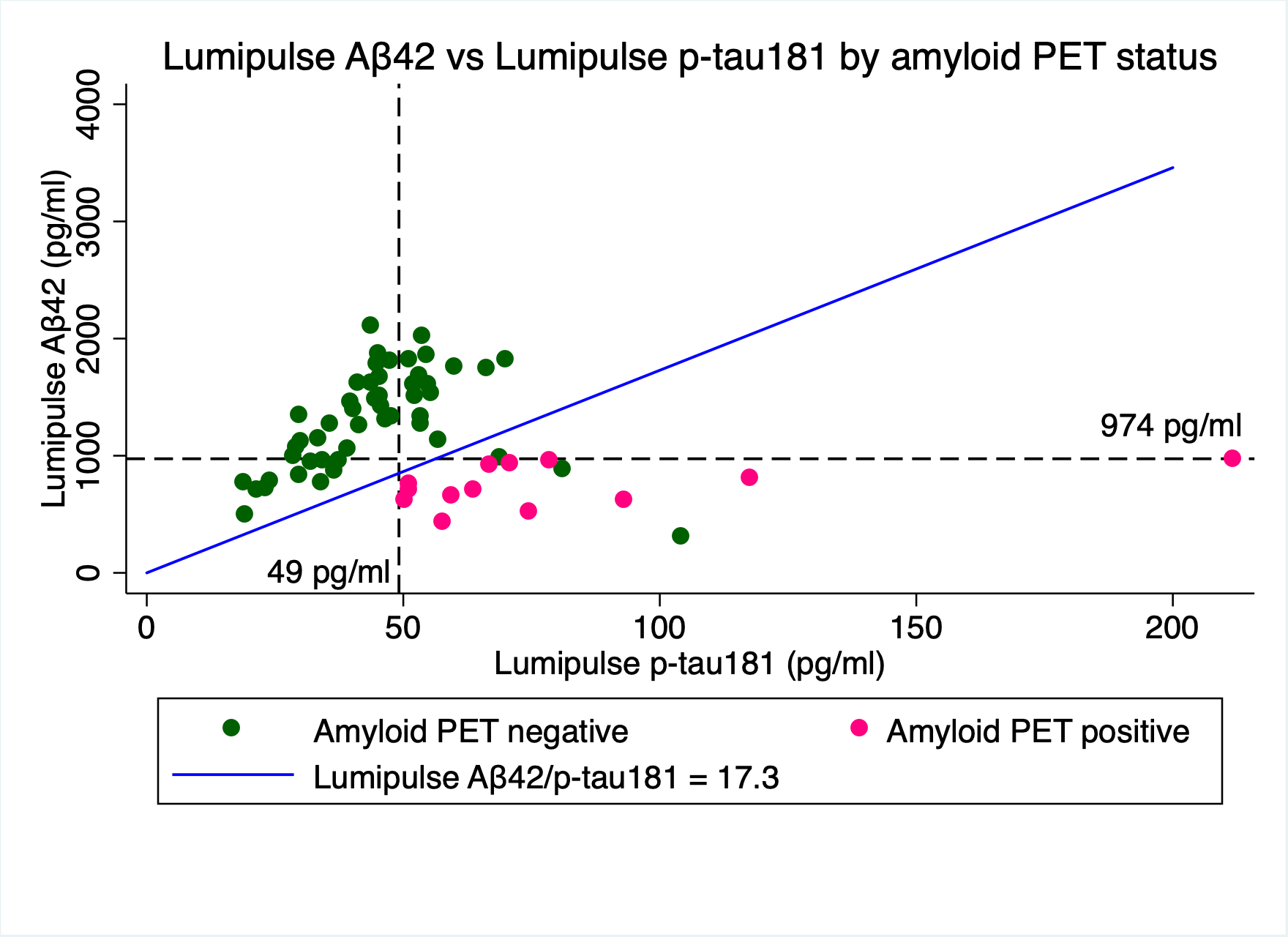 | F | 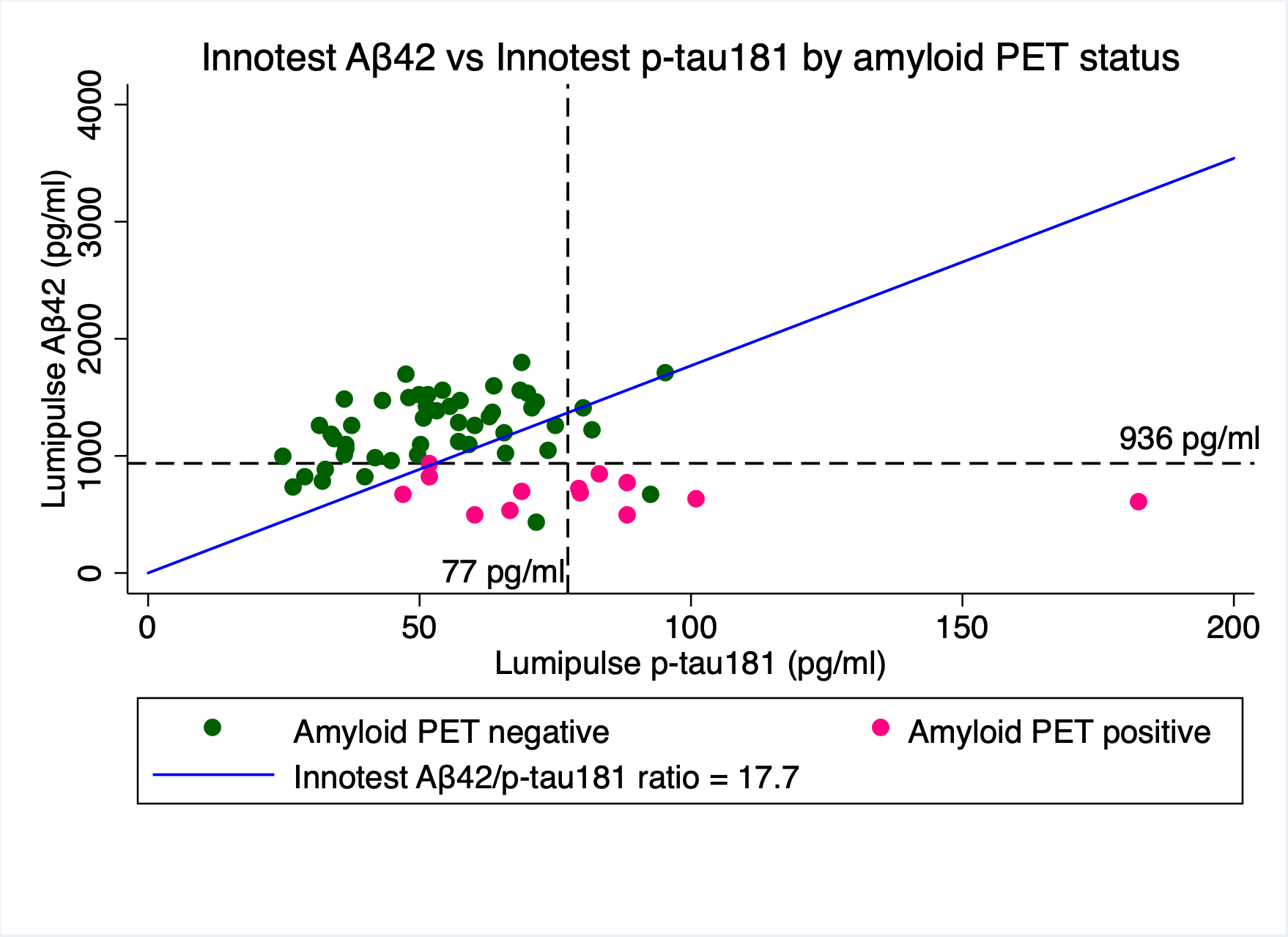 |

Blue lines indicate the ratio cut-point (Aβ42/Aβ40 ratio – Panel A and B; Aβ42/t-tau ratio – Panel C and D; Aβ42/p-tau181 ratio – Panel E and F). Dashed lines denote cut-points for the individual biomarkers that performed better than chance. Cut-points were determined by the Youden J index. The ^18^F-florbetapir amyloid PET SUVR cut-point was 0.61.

| A | 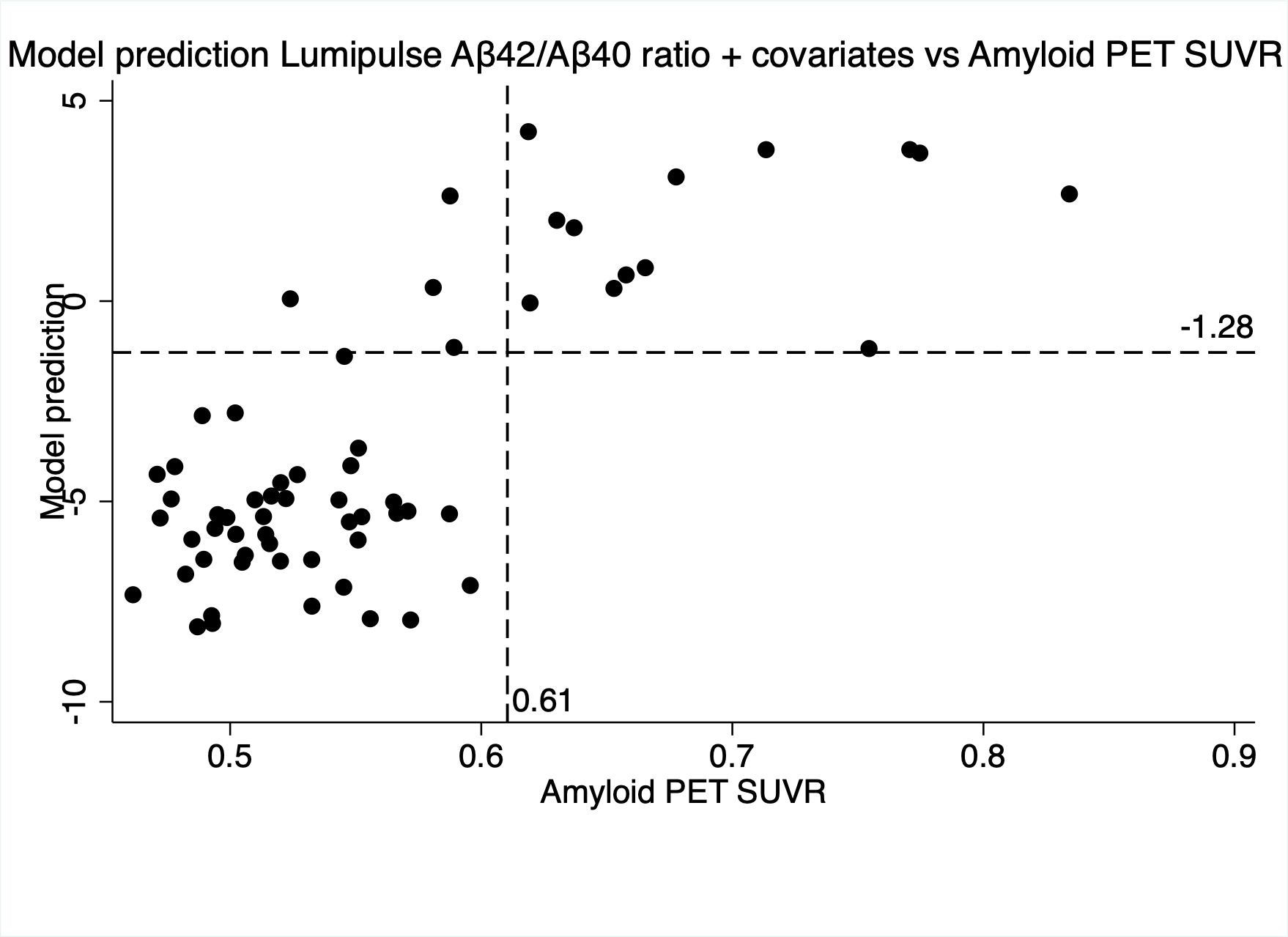 | B | 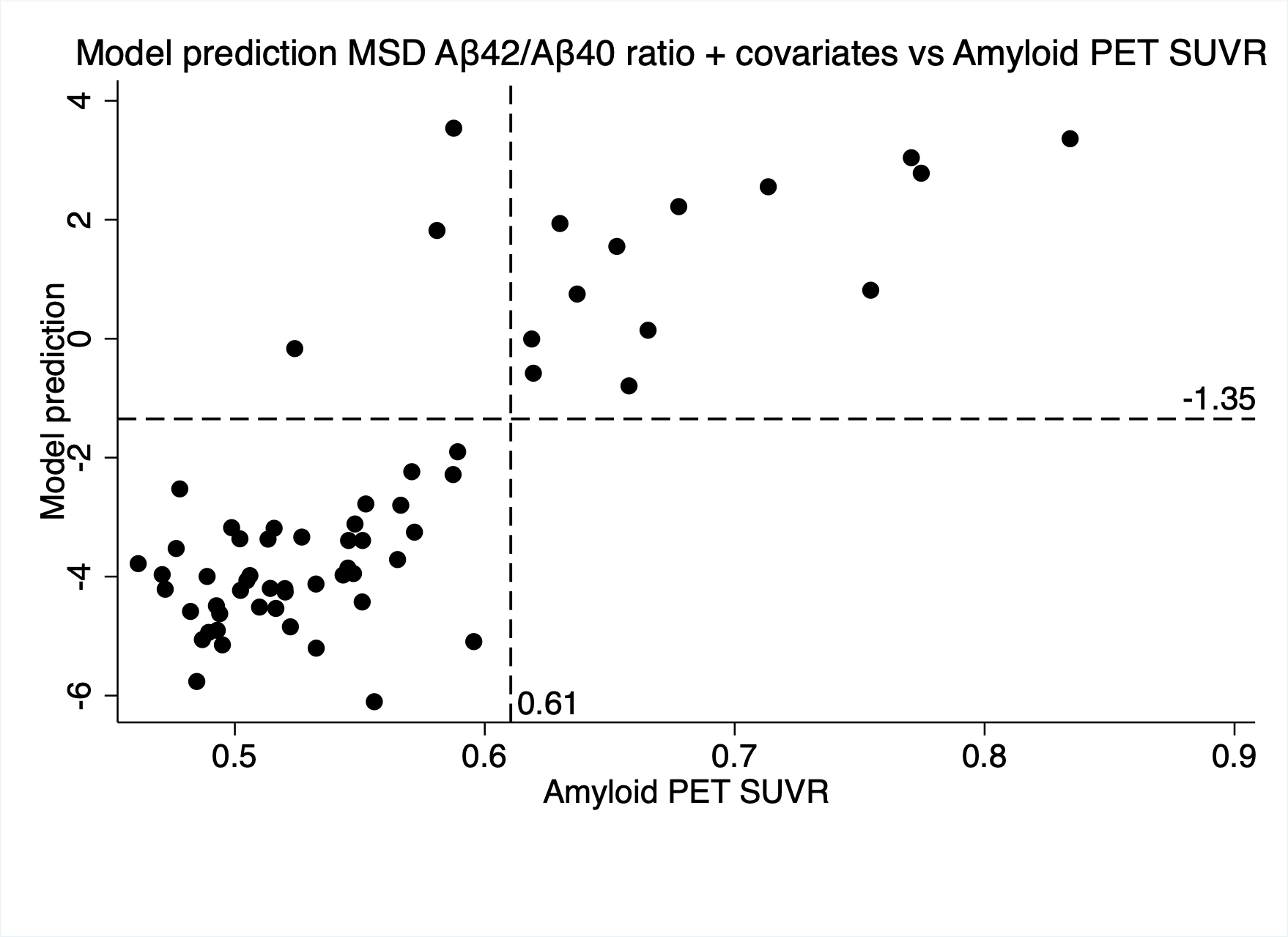 | C | 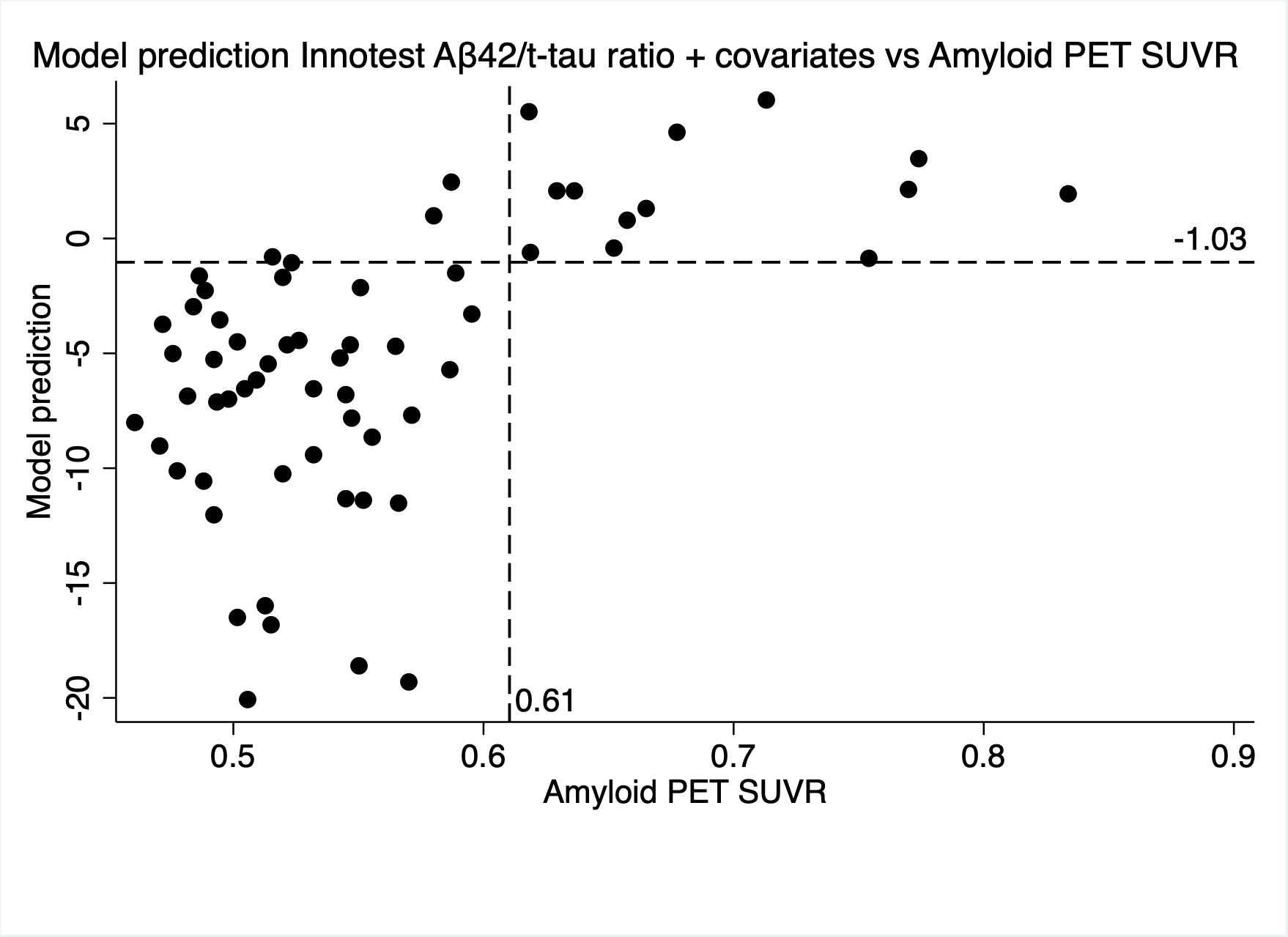 |
| --- | --- | --- | --- | --- | --- |
| D | 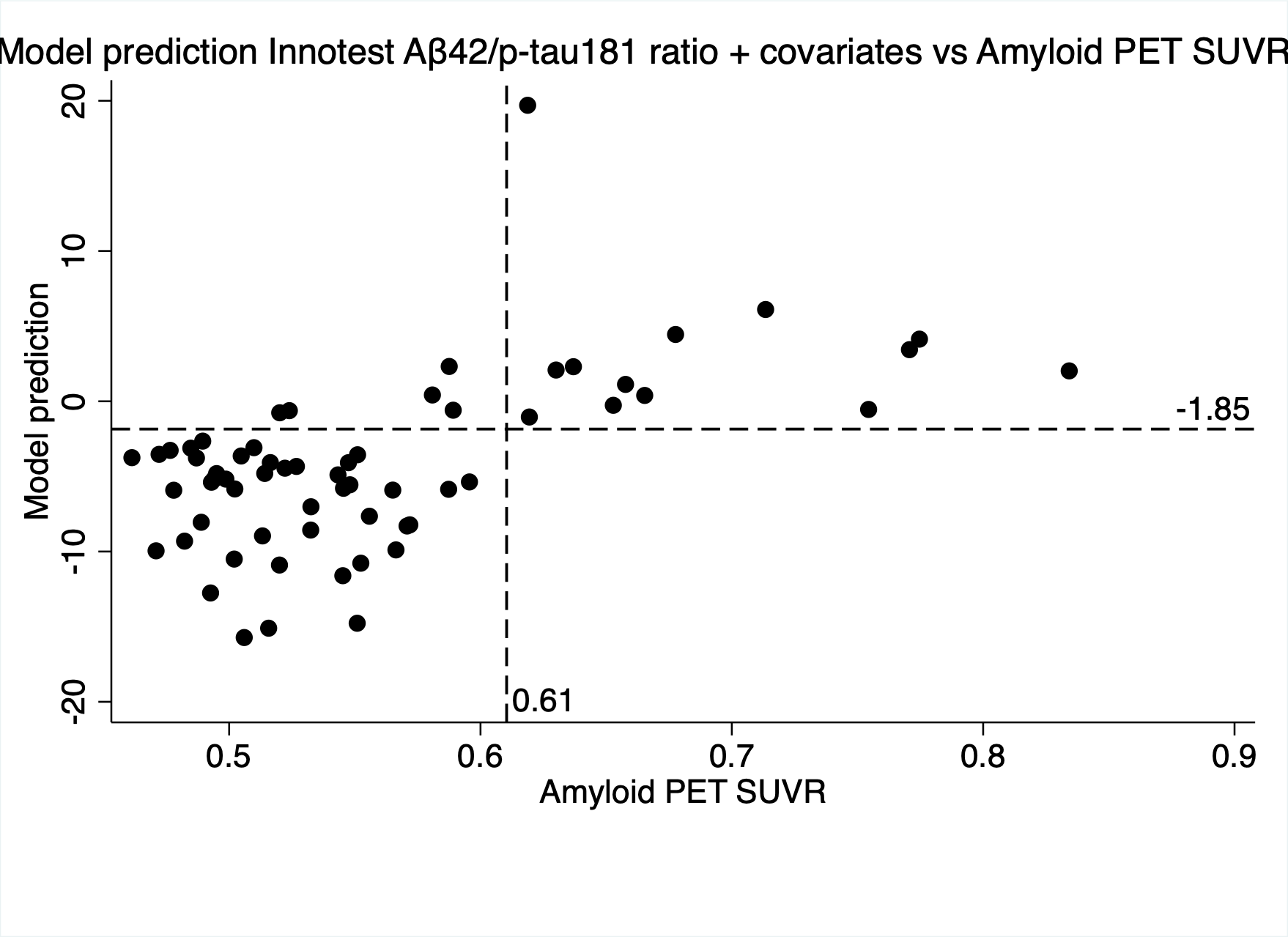 | E | 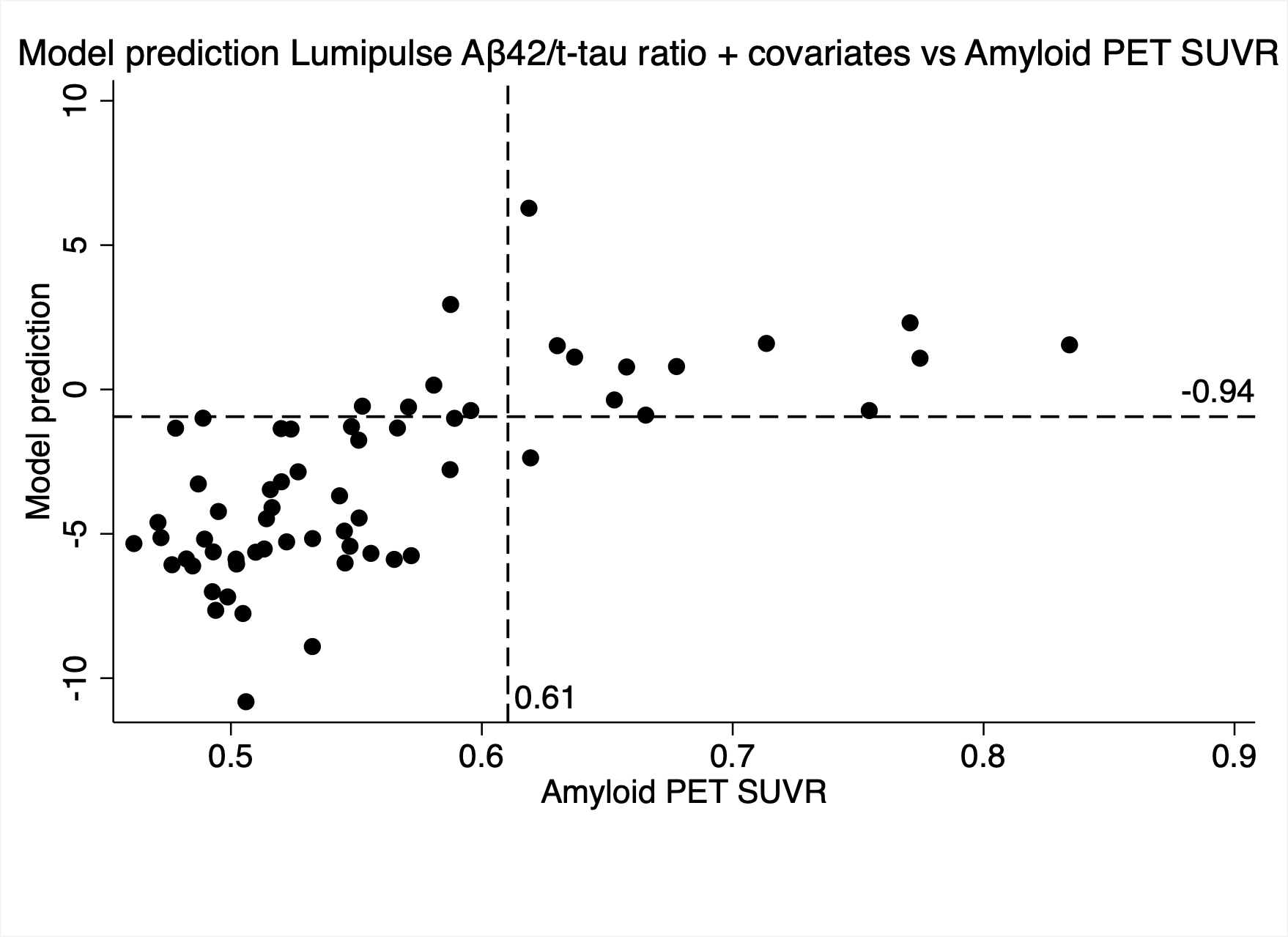 | F | 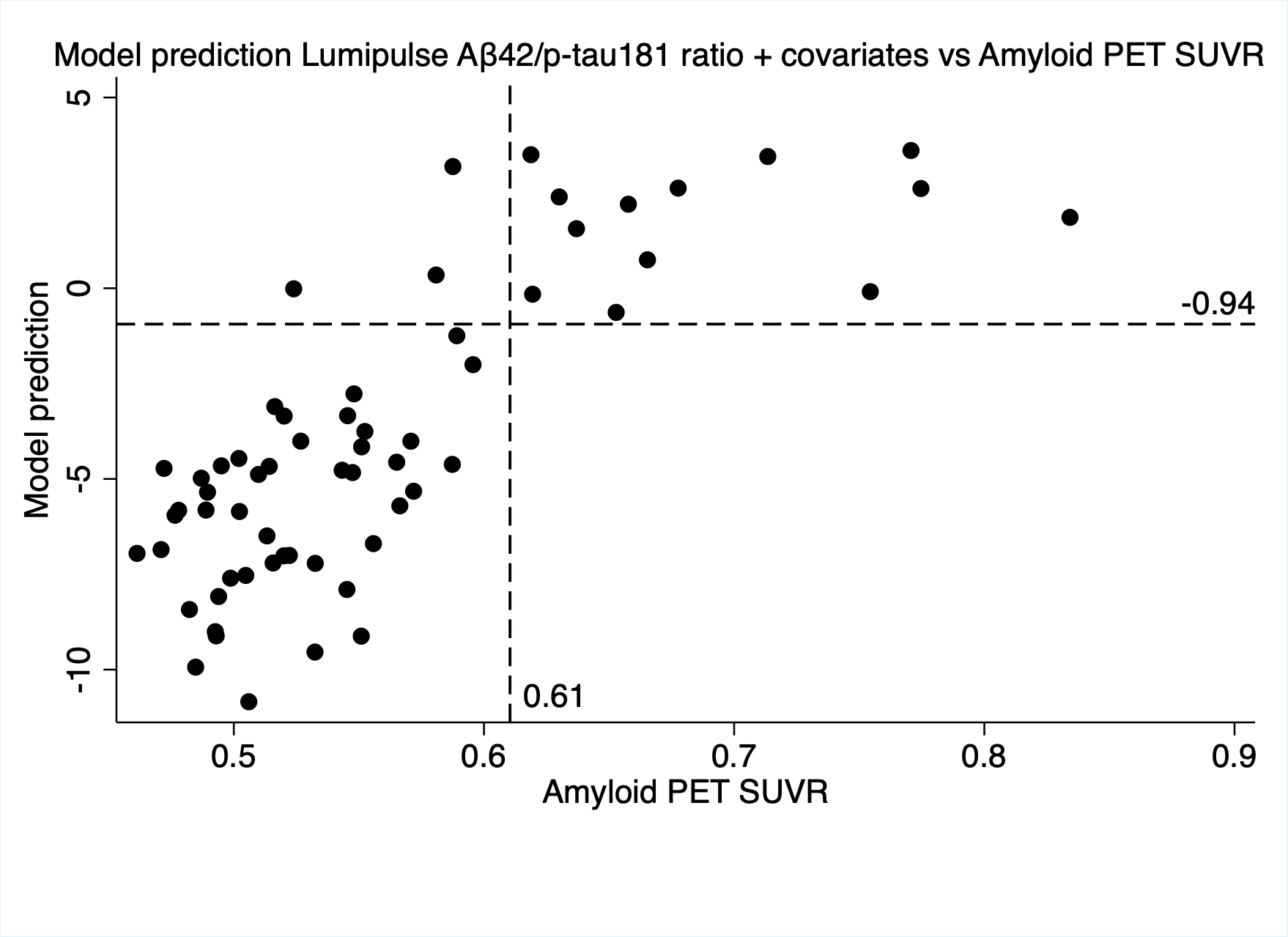 |

Supplementary figure 3: Scatter plots of predictions of models incorporating age, sex, APOE ɛ4 carrier status and CSF biomarker ratios (y axis) against SUVR (x axis), n=62

Dashed horizontal lines show the Youden’s index cut-points for the CSF ratios, above which an individual was classified as CSF–positive; dashed vertical lines show the ^18^F-florbetapir amyloid PET SUVR cut-point, to the right of which an individual was classified as amyloid PET–positive.
